# Light‐Driven ATP Regeneration in Diblock/Grafted Hybrid Vesicles

**DOI:** 10.1002/cbic.201900774

**Published:** 2020-04-07

**Authors:** Christin Kleineberg, Christian Wölfer, Amirhossein Abbasnia, Dennis Pischel, Claudia Bednarz, Ivan Ivanov, Thomas Heitkamp, Michael Börsch, Kai Sundmacher, Tanja Vidaković‐Koch

**Affiliations:** ^1^ Max Planck Institute for Dynamics of Complex Technical Systems Process Systems Engineering Sandtorstraße 1 39106 Magdeburg Germany; ^2^ Otto von Guericke University Process Systems Engineering Universitätsplatz 2 39106 Magdeburg Germany; ^3^ Jena University Hospital; Single-Molecule Microscopy Group Nonnenplan 2–4 07743 Jena Germany

**Keywords:** ATP synthase, bacteriorhodopsin, diblock polymers, energy conversion, graft polymers, permeability

## Abstract

Light‐driven ATP regeneration systems combining ATP synthase and bacteriorhodopsin have been proposed as an energy supply in the field of synthetic biology. Energy is required to power biochemical reactions within artificially created reaction compartments like protocells, which are typically based on either lipid or polymer membranes. The insertion of membrane proteins into different hybrid membranes is delicate, and studies comparing these systems with liposomes are needed. Here we present a detailed study of membrane protein functionality in different hybrid compartments made of graft polymer PDMS‐g‐PEO and diblock copolymer PBd‐PEO. Activity of more than 90 % in lipid/polymer‐based hybrid vesicles could prove an excellent biocompatibility. A significant enhancement of long‐term stability (80 % remaining activity after 42 days) could be demonstrated in polymer/polymer‐based hybrids.

## Graphical abstract

**Biocompatible functionality**: EF_O_F_1_ ATP synthase and bacteriorhodopsin are reconstituted in hybrid vesicles based on grafted polymer PDMS‐g‐PEO and diblock polymer PBd‐PEO. The usage of hybrid vesicles instead of liposomes significantly increases the long‐term stability of the ATP regeneration module.

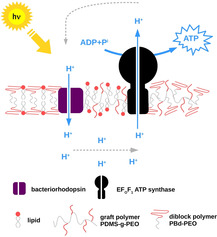

## Introduction

Artificial organelles are fascinating building blocks to design functional biomimetic devices and systems as well as cell‐like entities in the field of bottom‐up synthetic biology. In addition, they may contribute to the better understanding of complex biological processes and principles of life.^1^ To this end, phospholipoproteins,^2^ protein‐polymer,^3^ and organic/inorganic hybrids^4^ were assembled in a controlled way in order to exhibit various cell‐like characteristics and functions such as cytoskeletal‐like structures,^5^ predatory behavior,^6^ and even self‐proliferation.^7^ Among other examples, artificial organelles for energy regeneration are recently receiving increasing attention. An overview of the existing approaches for energy regeneration, with major focus on artificial organelles is summarized in our recent article,^8^ whereby most of these approaches focused on ATP regeneration.

ATP is the main energy carrier in living organisms, fueling the majority of the energy consuming processes. It is generated through oxidative phosphorylation and photophosphorylation by the proton‐gradient driven enzyme F_O_F_1_‐ATP synthase. For mimicking natural photophosphorylation, a series of artificial systems have been constructed to capture the energy of light and move protons across the membrane.^9^ First reports for simple prototypes of systems, which combine light‐driven proton pumps with the F_O_F_1_‐ATP synthase in liposomes have been published already in the early 1970s,^10^ whereby the original motivation has been the development of *in vitro* models for the mechanistic understanding of F_O_F_1_‐ATP synthase. By variation of several different types of rhodopsins and F_O_F_1_‐ATP synthases as well as of the lipid composition, the productivity of these assemblies could be constantly improved.^10^, ^11^

In the framework of bottom‐up synthetic biology, new exciting possibilities for the combination of natural and synthetic based materials arise. In this regard, Choi and Montemagno^12^ reported the first successful incorporation of bacteriorhodopsin (bR) and ATP synthase from thermophilic *Bacillus PS3* (TF_O_F_1_) into polymersomes consisting of ABA triblock copolymers. Since then the interest in protein reconstitution into polymer‐based membranes increased.

According to the literature, one major benefit of polymersomes, compared to natural liposomes, is their enhanced functional durability.^13^ Here, the high mechanical stability and low proton permeability of polymersomes towards protons can be viewed as a positive factor, but also as a limiting feature in some applications where controlled permeation of species is required. A very promising method has recently emerged to overcome intrinsic limitations of both polymersomes and liposomes. The proposed approach employs mixed hybrid vesicles from both copolymers and lipids, enabling fine‐tuning of the membrane physical properties.^14^ A recent example demonstrates the functional incorporation of cytochrome bo_3_ quinol oxidase (bo_3_ oxidase) in such hybrid vesicles, consisting of diblock copolymer polybutadiene‐b‐poly(ethylene oxide) (PBd‐PEO) and 1‐palmitoyl‐2‐oleoyl‐sn‐glycero‐3‐phosphocholine (POPC).^13^ This combination results in a better biocompatibility of the hybrid membranes, compared to those of pure polymer membranes, alongside a remarkable enhancement in the functional lifetime of the enzyme.^13^ In other work, Jacobs et al.^15^ showed an increased folding of a mechanosensitive channel protein during cell‐free expression in PBd‐PEO hybrid vesicles compared to pure liposomes.

It is known that a key parameter for the functionality of transmembrane proteins is the lateral mobility within the membrane, which largely depends on its flexibility and fluidity.^16^ Therefore, another graft copolymer poly(dimethylsiloxane)‐graft‐poly(ethylene oxide) (PDMS‐g‐PEO) with higher fluidity^17^ and lower core thickness^18^ has been recently suggested for co‐assembly of F_O_F_1_‐ATP synthase and bo_3_ oxidase in hybrid vesicles.^19^ The functional incorporation of both enzymes in these hybrid vesicles has been demonstrated, but the functional durability has not been tested yet.

In the present work, light‐driven ATP regeneration has been studied in hybrid vesicles based on two different kinds of polymers, diblock copolymer PBd‐PEO and grafted polymer PDMS‐g‐PEO. The influence of membrane composition on the performance, proton permeability, reconstitution efficiency, long‐term stability and orientation of proteins in the membrane has been evaluated.

## Results

### Light‐driven ATP production in lipid vesicles

**Figure 1:**
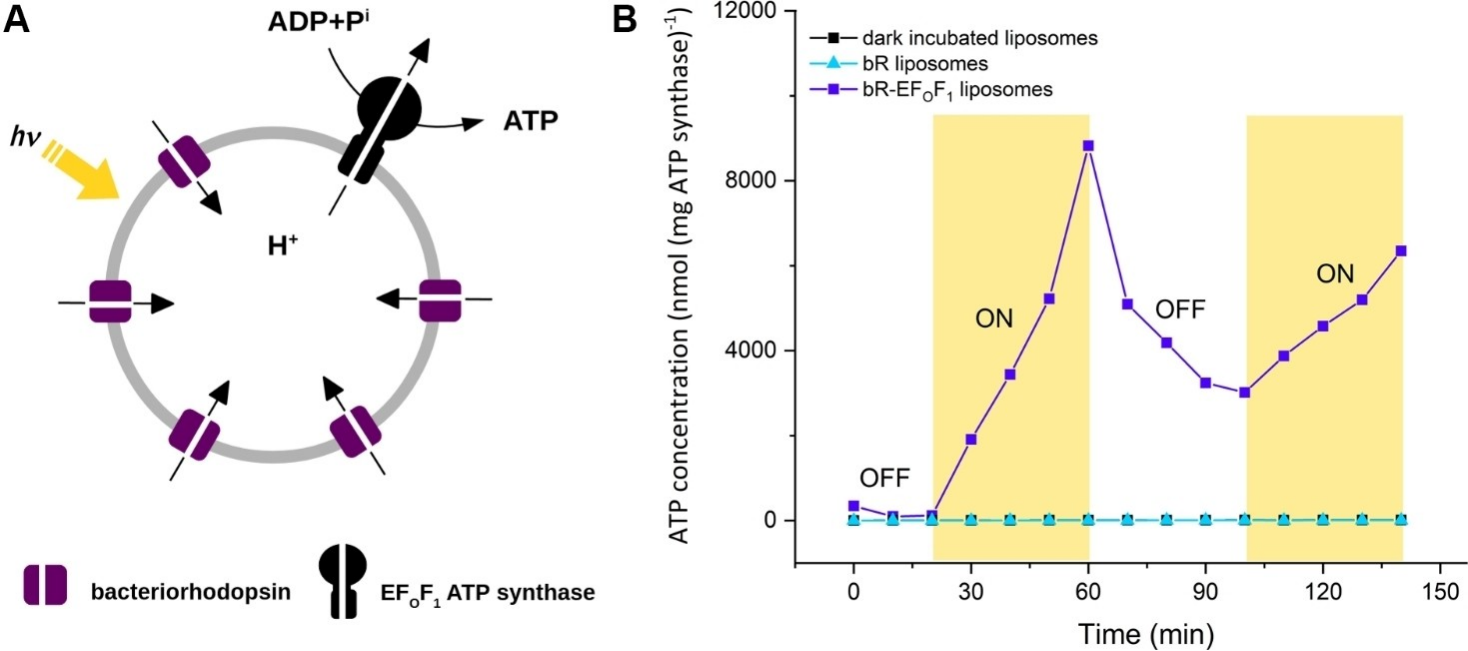
Light‐driven ATP synthesis in lipid vesicles (100‐150 nm). A) Schematic representation of the ATP‐generating system reconstituted with bacteriorhodopsin (bR) and F_O_F_1_‐ATP synthase. F_O_F_1_‐ATP synthase uses the electrochemical gradient generated by bR to synthesize ATP. B) Photoinduced ATP synthesis in bR‐EF_0_F_1_ liposomes through on‐off cycles of light, in the absence of light (dark incubated liposomes), and in liposomes containing only bR (bR liposomes).

An artificial light‐driven ATP regeneration module has been built through bottom‐up assembly of purified transmembrane proteins. Bacteriorhodopsin, a light‐driven proton pump, establishes a proton gradient to drive the synthesis of ATP by F_O_F_1_‐ATP synthase from *Escherichia coli* (schematically shown in Figure 1A). The reconstitution of both enzymes into phosphatidylcholine (PC) unilamellar vesicles is optimized for performance using a Triton X‐100 mediated reconstitution into preformed liposomes, similar to the method described by Fischer et al.11d The ATP production rate in the natural phospholipid environment is later used as a benchmark for the evaluation of biocompatibility of both transmembrane proteins in different hybrid vesicles.

The activity of both enzymes is quantified by measuring the produced ATP under illumination using the luciferin/luciferase assay. The calculation of ATP using the luminescence signal is described in the Supporting Information. Figure 1B represents the course of ATP production through on‐off cycles of light. The concentration of ATP increases under illumination and decreases again in the dark due to the absence of proton motive force (presumably due to ATP hydrolysis), which demonstrates ATP production triggered by light. As control ATP is measured in complete absence of light as well as in liposomes containing only bR. In both experiments no measurable increase of ATP is detected (Figure 1B).

We isolated both proteins according to a standard procedure^20^ and checked their purity using SDS‐PAGE analysis (Figure S1 in the Supporting Information). Figure S1A shows purified ATP synthase under denaturing conditions with distinct bands for subunit α, β, γ, δ, ϵ, a and b. Subunit c is known to be very weak on Coomassie‐stained SDS‐PAGE and is therefore barely visible. SDS‐PAGE of isolated bR shows a single band at the expected molecular mass of monomeric bR (Figure S1B).^21^ The absorbance spectrum has two characteristics peaks at 280 nm and 560 nm which correspond to the protein and the pigment, respectively. The ratio of the two amplitudes indicate a purity of around 98 %.^22^

The performance of ATP production depends on different factors, which makes a straightforward comparison with literature reports difficult. Besides the choice of reconstitution method and detergent, the origin and activity of the F_O_F_1_‐ATP synthase as well as the bR/ATP synthase ratio per liposome, have to be considered. The commonly used incorporation method is a co‐reconstitution of both enzymes using different detergents, mainly octylglucoside and Triton‐X‐100, to mediate the insertion of proteins into preformed liposomes.11e, 11f, 11g, 11h, 23 We decided for a Triton‐X‐100 mediated reconstitution, because octylglucoside has been reported to inactivate MF_O_F_1_‐and CF_O_F_1_‐ATP synthase11g and also led to much lower ATP production rates in our experiments (data not shown). For coupling bR with ATP synthase in lipid vesicles, different bR/F_O_F_1_‐ATP synthase ratios, ranging from 1 : 111e, 23c to 170 : 1,23a have been covered. The respective ATP production rates are increasing with the amount of bR (Figure S2), presumably caused by the establishment of higher driving forces.

### Light‐driven ATP production in hybrid vesiclesMembrane compositions

**Figure 2:**
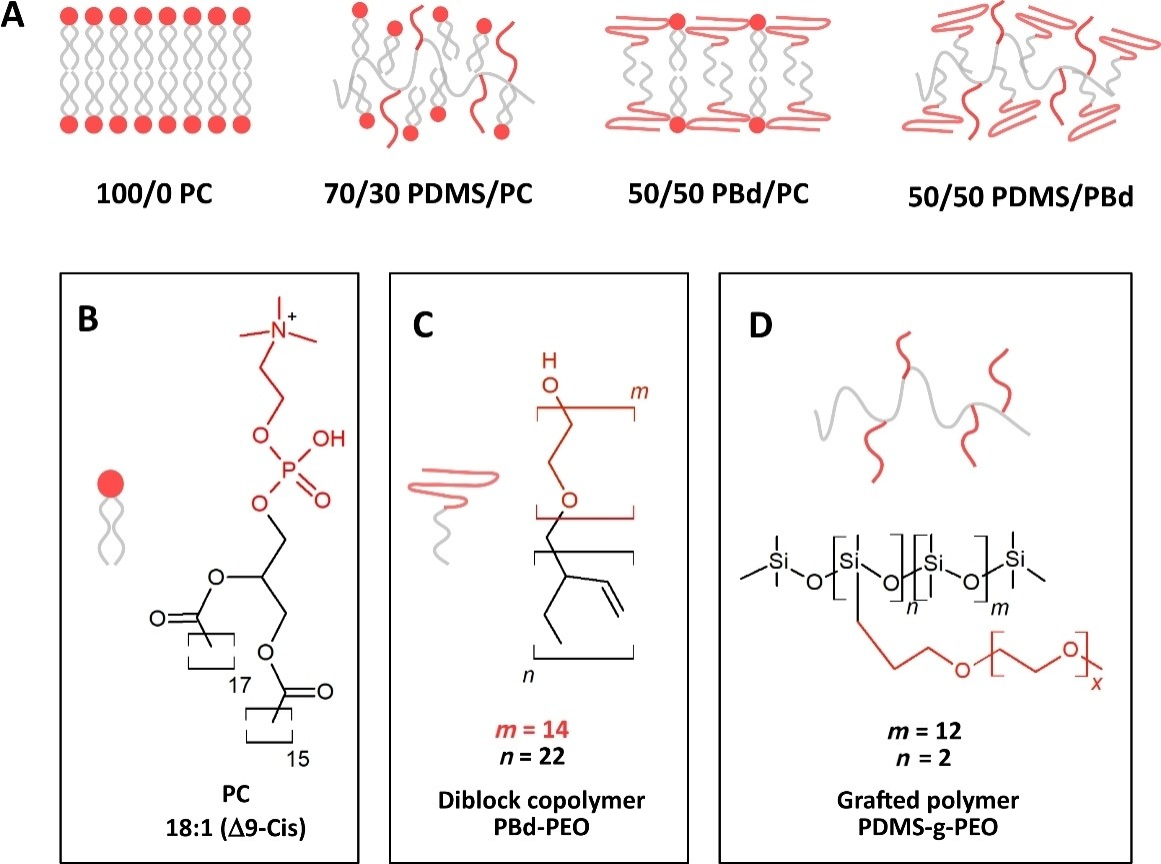
A) Schematic presentation of different membrane compositions: Pure PC vesicles (100/0 PC), hybrid vesicles made of 70 mol % PDMS‐g‐PEO (70/30 PDMS/PC), 50 mol % PBd‐PEO (50/50 PBd/PC) as well as a mixture of 50 mol % PDMS‐g‐PEO and 50 mol % PBd‐PEO (50/50 PDMS/PBd). Schematic representation of the nanoscale structures of B) phosphatidylcholine, C) diblock polymer PBd‐PEO and D) grafted polymer PDMS‐g‐PEO.

To study the influence of hybrid membranes on the performance of the ATP regeneration system, we prepared four different kinds of vesicles as schematically shown in Figure 2A. Pure soy PC vesicles (100/0 PC) are used as a benchmark for enzyme activity in other compartments. The other three types of vesicles are all hybrid vesicles made of PC lipids (Figure 2B) and two types of polymers with different fluidities and structure: 1) A comb‐type siloxane surfactant, poly(dimethyl siloxane)‐g‐poly(ethylene oxide) (PDMS‐g‐PEO),^19^ and 2) a diblock copolymer, polybutadiene‐b‐poly(ethylene oxide) (PBd‐PEO)^13^, ^14^, ^24^ (Figure 2C, D). Both polymers have the same hydrophilic block (PEO), while the hydrophobic block as well as the polymer architecture differs.

PDMS‐g‐PEO (denoted hence PDMS) is known to form vesicular structures with a membrane thickness of around 5 nm, close to that of natural lipid membranes. This grafted copolymer self‐assembles into monolayers, flexible enough to incorporate complex membrane proteins^25^ and forms well‐mixed membranes above 70 mol % polymer. Therefore, we decided to investigate hybrids made of 70 mol % polymer (70/30 PDMS/PC) in this study.

PBd‐PEO (denoted hence PBd) in contrast, is a diblock copolymer that self‐assembles into bilayers. We chose the 1.8 kDa polymer PBd_22_‐b‐PEO_14_ as these polymer chains are expected to have similar membrane thickness as lipid vesicles, estimated ∼4–5 nm, thereby minimizing the hydrophobic mismatch between polymer and lipid.^15^ PBd polymer with higher molecular size, as for example PBd_37_‐PEO_22_, forms membranes with increased thickness and the mismatch between lipid and polymer thickness presumably results in lower biocompatibility of hybrid vesicles. PBd is known to form homogeneous, well‐mixed hybrid vesicles with POPC within the whole range of compositions.^26^ We decided to study hybrid vesicles made of 50 mol % polymer (50/50 PBd/PC) as this composition has been shown superior to be the best with inserted bo_3_ oxidase.^13^, 24a

Hybrid vesicles made of PDMS‐g‐PEO and PBd‐PEO form well‐mixed vesicles, bearing the strength and toughness characteristics of pure PBd‐PEO vesicles as recently shown by Gaspard et al.^27^ As both polymers have different membrane permeabilities and fluidities, a combination of both may allow for systematic, application‐specific tuning of membrane fluidity and permeability. The functional reconstitution of proteins in hybrid membranes made of these two polymers could offer new possibilities for the design of nanoreactors, biosensors or artificial organelles. In this respect, our fourth vesicle system is based on a mixture of 50 mol % PDMS and 50 mol % PBd (50/50 PDMS/PBd).

### Reconstitution procedure

**Figure 3:**
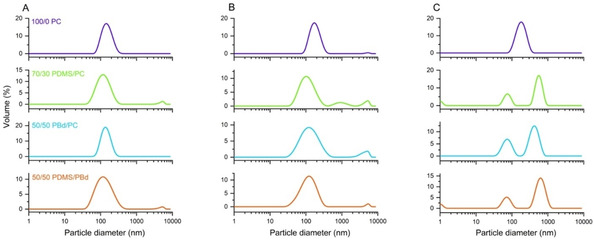
Size distribution of vesicles made of pure 100/0 PC, 70/30 PDMS/PC, 50/50 PBd/PC and 50/50 PDMS/PBd. A) Sizes of vesicles after extrusion through 100 nm pores. B) Size distribution of detergent‐treated vesicles prior to reconstitution. C) Sizes of vesicles after reconstitution and removal of detergent using bio beads.

For insertion of transmembrane proteins in hybrid compartments, we decided for a Triton X‐100 mediated reconstitution into preformed vesicles similar to method described for reconstitution in liposomes. Hybrids are prepared by film rehydration method, followed by extrusion through 100 nm pores. The size distribution profiles after extrusion as measured by dynamic light scattering (DLS) (Figure 3A) show that all vesicles are uniform in size with an average diameter of 141 nm for 100/0 PC, 116 nm for 70/30 PDMS/PC, 136 nm for PBd/PC and 121 nm for 50/50 PDMS/PBd.

Turbidity profiles for each membrane composition are taken (Figure S3) to determine the amount of Triton X‐100 necessary to dissolve the vesicles at the intermediate step of solubilization. This point is proposed by Rigaud and colleagues11g, 11h, 28 for optimal membrane protein insertion when using Triton X‐100 as detergent.

PC liposomes are solubilized with 0.3 % Triton X‐100, while all hybrid vesicles are solubilized with remarkably lower Triton X‐100 concentration of 0.06 %. DLS data after treatment with detergent are taken prior to reconstitution (Figure 3B) and indicate that vesicles remain intact and are existent as detergent‐saturated membranes. ATP synthase and bR are added to the vesicles as detergent‐solubilized monomeric proteins aiming to a theoretical protein‐per‐liposome ratio of approximately 1 ATP synthase and 28 bR molecules. After 1 h of incubation in the dark, the detergent is removed by addition of bio beads. The amount of bio beads has been chosen in a way to allow for rapid detergent removal and thus to avoid self‐aggregation of ATP synthase.

DLS data after detergent removal (Figure 3C) show that the addition of bio beads has a critical effect on the hybrid size distribution. While PC liposomes retain their uniform size, all hybrid vesicles split in two distinct fractions – a smaller fraction of around 75 nm and a larger fraction of around 400–600 nm in diameter. Even though all vesicles are treated in the same way with bio beads, hybrid vesicles seem to behave differently. This might be explained by the slow, viscous dynamics of the polymers which might influence vesicles formation during detergent removal.^13^ Another interpretation would be the formation of non‐vesicular assemblies or large aggregated structures and will be further addressed in the discussion. In the present study, both populations of bigger and smaller sized vesicles are used for further measurements without separation.

**Performance**

**Figure 4:**
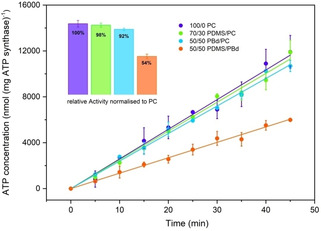
Light‐driven ATP synthesis in lipid and hybrid vesicles. The activity is determined by linear regression. The inset bars show the activity normalized to pure PC liposomes. All error bars represent the standard error of three independent measurements.

Light‐driven ATP production in hybrid vesicles is quantified by using the luciferin‐luciferase assay as described above and the activity is compared with the activity in pure lipid vesicles (Figure 4). The results show a fairly small reduction in activity for 70/30 PDMS/PC (98 % activity) and 50/50 PBd/PC (92 % activity), but more considerable reduction in activity for 50/50 PDMS/PBd hybrids (54 % activity). Previous work integrating bo_3_ oxidase and ATP synthase in 70/30 PDMS/PC hybrids^19^ confirmed bo_3_ oxidase activities of 93 % compared to liposomes, while the overall ATP production activity was around 56 %. One reason for this comparably lower performance might be a deviant reconstitution procedure using octylglucoside as detergent, since as mentioned above, octylglucoside has been shown to inactivate ATP synthase.11g, 11h Khan et al.^13^ reached activities of around 80 % when reconstituting bo_3_ oxidase in hybrid vesicles made of 50 % PBd.

The lower activity of our system in 50/50 PDMS/PBd hybrids can be explained by the fact that bio‐functional lipids are missing in this composition. In general protein activity has been shown to decrease with decreasing lipid content.^13^, ^19^

As mentioned above, the concentrations of enzymes are chosen in a way aiming for a theoretical protein‐per‐vesicle ratio of approximately 1 ATP synthase (0.1 μM) and 28 bR molecules (2.9 μM). Earlier studies11f, 11g, 11h have shown that a 1 : 1 ATP synthase to bR ratio is not enough to drive ATP synthase efficiently. Figure S2 represents the dependence of the ATP production rate on the concentration of bR in our setup. The results indicate that the ATP production is still limited by the amount of bR when using a protein‐per‐vesicle ratio of 1 : 28 (ATP synthase/bR). The functionality of bR is therefore relevant for the overall efficiency of the ATP production module. In this regard we investigated the influence of different membrane compositions on the activity of bR reconstituted alone in the absence of ATP synthase.

### bR reconstitution efficiency and proton pumping activity

**Figure 5:**
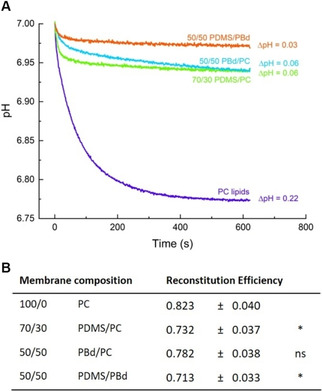
A) Proton pump activity of bR in liposomes and different hybrid vesicles as measured by pH change upon irradiation with green light. pH change is detected by encapsulated pyranine. Results show the middle value of at least three separate measurements. B) Reconstitution efficiency of bR in different compartments. *P≤0.05, not significant (ns) *P*>0.05 (*P* values are generated by unequal variance t‐test (Welch's test) for comparison of each hybrid membrane composition to the lipid vesicle sample). *n*=3; errors represent the standard error of the mean (SEM).

For measurement of bR proton pumping activity in different compartments, we prepared PC liposomes and hybrid vesicles in the presence of 8‐hydroxyprene‐1,3,6‐trisulfonic acid (pyranine), a pH‐sensitive dye. bR is then reconstituted following the co‐reconstitution method described above. After removal of detergent using bio beads, the vesicles are passed over a gel filtration column. The gel filtration enables, on the one hand, to remove non‐encapsulated pyranine, and on the other hand, to separate non‐reconstituted bR. The reconstitution efficiency could be calculated directly by the absorbance rate at 560 nm before and after gel filtration (Figure 5B). Results indicate highest reconstitution efficiency in liposomes (82 %) and slightly lower reconstitution efficiency (71‐78 %) in hybrid vesicles. A significant difference (*) in reconstitution efficiency compared to liposomes is only indicated for 70/30 PDMS/PC and 50/50 PDMS/PBd hybrids.

Proton pumping rates of bR upon green light irradiation are shown in Figure 5A for different membrane compositions. Since the relative absorbance ratio of pyranine at 450 and 405 nm (*A*_450_/*A*_405_) is dependent on the proton concentration, internal pH in vesicles can be determined by reading the characteristic absorbance maxima at 450 and 405 nm. The conversion from *A*_450_/*A*_405_ to pH is performed using a calibration curve (Figure S4).

A rapid acidification during the initial seconds of illumination is followed by a negligibly small change in pH. This progressive decrease as the gradient is established can be attributed to the back‐pressure effect of ΔpH.^29^ The maximal steady‐state ΔpH (0.22) is found in pure PC lipid vesicles and remarkably lower values are detected in lipid/polymer‐mix hybrid vesicles. One possible reason can be the presence of two populations of lipid/polymer‐mix vesicles (Figure 3) compared to a single lipid vesicle population. The lowest steady‐state ΔpH (0.03) is seen in 50/50 PDMS/PBd. The initial pumping rate in 70/30 PDMS/PC vesicles is higher compared to that in 50/50 PBd/PC vesicles, while the steady state ΔpH (0.06) remains the same. The investigation of pumping rates depending on the amount of pumping units (Figure S6) show a similar effect. Initial rates of proton pumping are increasing with the protein content, while the steady‐state ΔpH stays constant. The reason why the total proton uptake, in this case, remains unchanged, might be either explained by back‐pressure effect of ΔpH or by passive proton permeability of the membrane.^29^ In the case of hybrid vesicles, results might indicate that more active pumping units are present in 70/30 PDMS/PC hybrids compared to 50/50 PBd/PC hybrids, but that back‐pressure effects and passive proton permeability will lead to a similar steady state ΔpH. Another interpretation would be a slower turnover rate of bR in 50/50 PBd/PC hybrids due to the higher viscous environment. It has to be mentioned that the slope of the curve of 50/50 PBd/PC vesicles indicate that the actual steady‐state ΔpH is not achieved after 600 s. Higher ΔpH might be reached if the experiment have been run for longer.

In order to estimate the number of active pumping units, the orientation of bR in different membranes is investigated.

### bR orientation in different membranes

**Figure 6:**
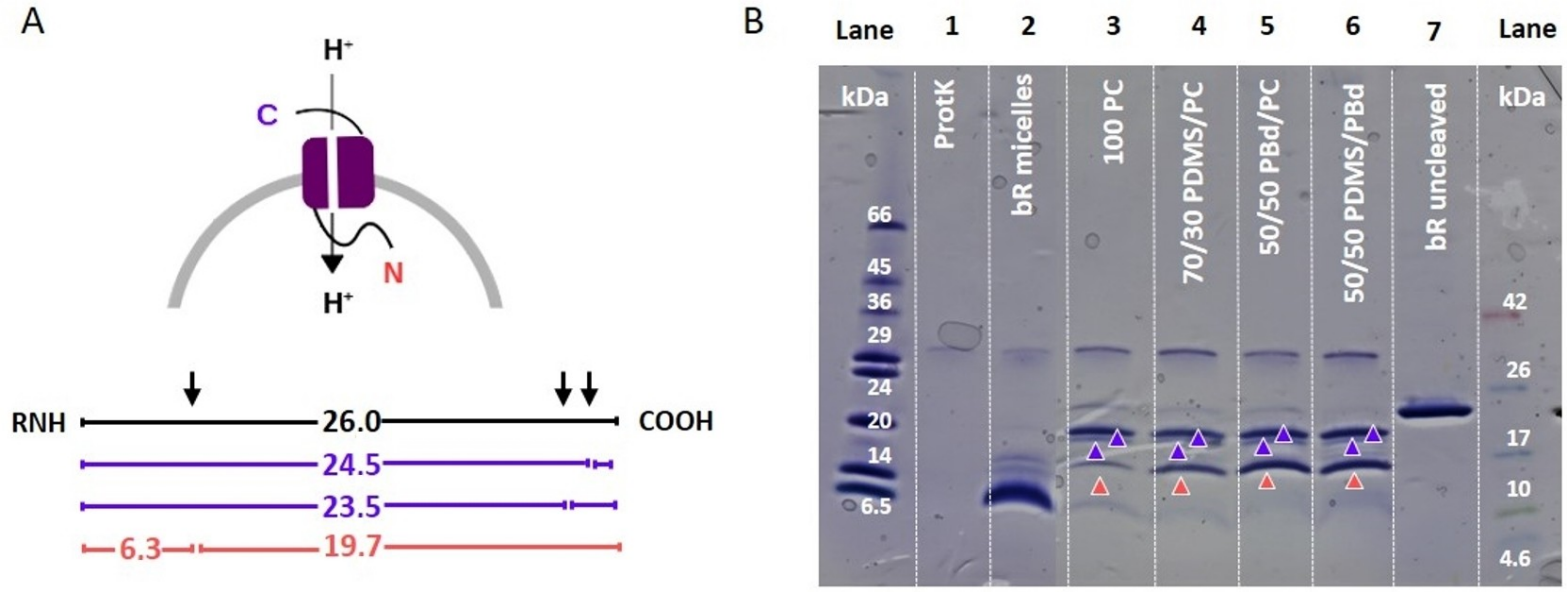
Proteolytic cleavage of reconstituted bR with proteinase K (ProtK) shows mixed orientation in all lipid and hybrid vesicles. A) Expected sizes of proteolytic fragments for ProtK digestion of bR when the N‐terminal (red values) or C‐terminal (violet values) is exposed to the bulk solution. B) SDS‐PAGE gel analysis of the digestion products. Lane 1: band specific for ProtK enzyme only; lane 2: digestion product of not reconstituted bR; lanes 3–6: digest patterns for different lipid/hybrid vesicles containing solubilized bR; lane 7: undigested bR in lipid vesicles.

The orientation of bR in the membrane is one of the major factors that determines the protein pumping activity. While ATP synthase is mainly orientated with the hydrophilic head outwards,^30^ the orientation of bR is more random. We determined bR orientation in different compartments by using a proteolytic digestion assay with a nonspecific serine protease (proteinase K, ProtK) similar to the approaches of Gerber^31^ and Kalmbach^32^ (Figure 6). When a membrane protein is incorporated into vesicles, the membrane shields the core of the protein and only the hydrophilic residues that form loop regions remain exposed to the solution. Thus, properly folded and embedded bR only has a few sites accessible to proteolysis. These cleavage sites are located asymmetrically on both sites of the membrane.^33^ Consequently, ProtK‐induced cleavage on different sides of the membrane produces a distinct set of protein fragments (Figure 6A). These fragments can be distinguished by SDS‐PAGE analysis of digestion products (Figure 6B). bR‐containing samples that have not been exposed to ProtK show an intensive band at around 22 kDa (Figure 6B, lane 7).

The discrepancy between the molecular size determined in the gel (22 kDa) and the theoretical mass of bR (26.8 kDa)^33^, ^34^ can be explained by a phenomenon known as gel shifting. Membrane proteins are often not completely denatured by SDS and therefore migrating with smaller molecular size.^35^

ProtK itself (Figure 6B, lane 1) shows a single band at around 34 kDa. Not reconstituted bR in micelles (Figure 6B, lane 2) after cleavage with ProtK reveals a set of digestion products between 5 and 16 kDa. In this case, a majority of small digestion products is expected as ProtK can access bR unrestricted when there is no membrane shielding. We found the digestion products of proteo‐vesicles (Figure 6B, lane 3–6) to be different from the products of digestion of bR in micelles.

SDS‐PAGE shows 3 distinct bands for all membrane compositions at approximately 16, 19 and 21 kDa. The 16 kDa band (Figure 6B, orange arrow) can, under the assumption of gel shifting, be related to the N‐terminal protein fragments. The 19 and 21 kDa bands (Figure 6B, violet arrows) in contrast can be related to the C‐terminal fragments with a theoretically supposed size of 23.5 and 24.5 kDa.^31^ These results show that there is no uniform bR orientation in all vesicles. Anyway, a slightly better orientation is indicated in 100/0 PC, as the band at 16 kDa (orange arrow) is somewhat lighter compared to that of hybrid vesicles. An almost one‐sided orientation of bR could be only achieved in our lab when reconstituting bR in form of membrane patches (Figure S7). In these experiments bR is not solubilized with Triton prior to reconstitution. The purple membrane arranges bR in a 2D hexagonal crystalline lattice and contains 75 % bR embedded in 25 % lipid.^36^ In these patches all bR is orientated uniform. Moreover no additional detergent is added to the reconstitution by solubilized bR itself. As the patches stay partially intact a more uniform orientation might be favored.

Besides protein orientation, passive proton leakage through the membrane is an important parameter that influences attainable ΔpH values established by bR.^29^

### Passive proton permeability of lipid and hybrid vesicles

**Figure 7:**
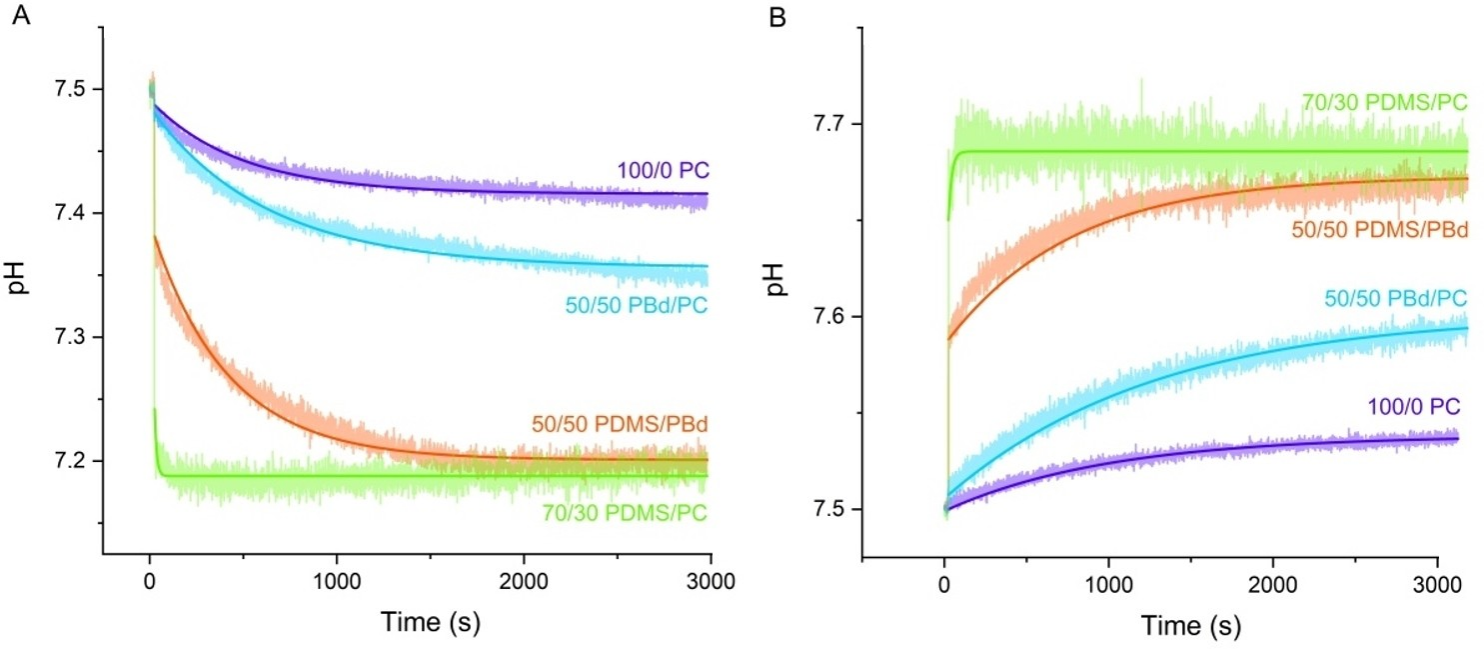
Proton permeability of vesicles as measured by pH change after addition of A) 2.4 mM HCl and B) 1.6 mM NaOH to the outer solution of vesicles. pH change is detected by encapsulated HPTS. The solid lines represent the simulations as predicted by the model described in the Supporting Information.

bR proton pumping experiments alone indicate much higher activity in PC lipids compared to hybrid membranes, which could be not completely explained by the reconstitution efficiency or the orientation of bR in the membrane. Another factor influencing the magnitude of ΔpH is the passive proton permeability of the membrane. We followed the kinetics of pH change inside different lipid/hybrid vesicles upon addition of HCl/NaOH to the outer solution in order to determine the permeability coefficient (*P*) for different membrane compositions (Figure 7). All measurements are conducted in PIPES buffer adjusted to pH 7.5 with KOH (50 mM KOH) according to Paxton et al.^37^ Valinomycin is added to prevent the build‐up of electrostatic potential differences, which are counteracting to the passive proton flux.

The pH inside the vesicles changed rapidly with the addition of NaOH/HCl, and then increased/decreased much more gradually. All time courses show a fast initial jump directly after adding acid/base. This jump is more significant in PDMS hybrids (70/30 PDMS/PC, 50/50 PDMS/PBd) compared to a more gradual increase in the case of PBd hybrids (50/50 PBd/PC) or liposomes (100/0 PC). The final pH (after 3000 s) also varies depending on the vesicle composition. In the case of 50/50 PDMS/PBd and 70/30 PDMS/PC, final pH values are lower (HCl) or higher (NaOH) and addition of nigericin (data not shown) induces no further equilibration of outer and inner pH. In contrast, the addition of nigericin to 50/50 PBd/PC and 100/0 PC vesicles causes a rapid pH jump.

The two‐stage pH change described above is well known.^29^, ^37^, ^38^, ^39^ The initial pH jump inside the vesicles upon addition of acid/base has been attributed by some literature to a rapid H^+^/OH^−^ permeability, which results in an uncompensated build‐up of charge. The H^+^/OH^−^ diffusion then slows down to a rate limited by the permeability of charge‐compensating co‐ions or counter‐ions.^37^, ^39^, ^40^ Other reports in contrast^29^ suppose leakage of small amounts of pyranine in the outer solution. Paxton et al.^37^ evidenced that this initial fast jump is critically influenced by the choice of buffer. In accordance to this, we also observed stronger initial jump with decreasing buffer capacity (data not shown), which is predicted by our mathematical model (Figure S9) described below.

To check the influence of valinomycin on the proton flux we performed measurements under the same conditions without adding valinomycin (Figure S8). As expected the proton flux slows down due to the build‐up of electrostatic potential differences. It can be seen that also the initial jump is decreasing in the absence of valinomycin. Interestingly, this effect seems to be more relevant when adding HCl instead of NaOH. In general, it has to be mentioned that the activity of ionophores is not yet investigated in detail for all hybrid vesicles used here. The activity of valinomycin in these polymer‐based membranes is just an assumption.

Different methods for determination and calculation of permeability coefficients have been reported.^29^, ^37^, ^38^, ^41^ Kuyper et al.38a for example derived the proton permeability coefficient by double exponential fitting under consideration of the vesicle size. In this study, we developed a model to describe the permeability of different vesicle membranes. In addition, we used the method recently described by Paxton et al.^37^

The mathematical model for the description of membrane permeability considers chemical as well as electrochemical driving forces for proton diffusion through the membrane^42^ (Method 1 in the Supporting Information). This model takes the size of the vesicles and the buffer capacity into account. The model cannot describe the initial fast jump, except by introducing unrealistically large values of permeability coefficients (e. g., the case of 70/30 PDMS/PC) or by accepting higher fitting errors at initial times (e. g., 100/0 PC; Figure 7, solid lines).Therefore, all data are fitted without taking the initial jump into account. A detailed description of equations and model parameters can be found in Figures S9 and S10.

For comparison we also used the method for determination of permeability coefficients recently described by Paxton et al.^37^ Permeability coefficients are calculated using the data during the first 200 s of the reaction (Method 2, Figure S11). Thereby the assumption of linear pH change during this period is made, which is not supported by experimental evidences for 50/50 PDMS/PBd and 70/30 PDMS/PC systems due the two‐stage pH change described above. Therefore, no permeability coefficients were calculated for these two cases. The proton permeability coefficients yield by both methods are summarized in Table 1.

**Table 1:** Permeability coefficients for different membrane compositions as yield by model simulation (Method 1) and coefficients calculated according to Paxton et al.^37^ (Method 2).

Membrane composition		P×10^9^ (cms^−‐1^) Method 1	P_OH−_×10^10^ (cms^−‐1^) Method 2	P_H+_×10^10^ (cms^−‐1^) Method 2
100/0	PC	1.88	3.4	9.4
70/30	PDMS/PC	1100	–	–
50/50	PBd/PC	2.41	6.2	19.8
50/50	PDMS/PBd	2.96	–	–

Permeability coefficients for 100/0 PC and 50/50 PBd/PC vesicles determined by both methods show the same trend. Pure liposomes have the lowest proton permeability and slightly higher permeabilities are calculated for PBd/PC hybrids. The observation that vesicles with intermediate lipid/PBd‐PEO ratios tend to be surprisingly more permeable to ion transport than pure lipid or pure polymer vesicles, has been made earlier by Paxton and colleagues.^37^ They used the larger PBd_37_‐PEO_22_ polymer and suggested that the higher permeability likely arises from the size mismatch between lipid and polymer. In the present case, lipid and PBd_22_‐PEO_14_ polymer have a similar size. P values determined here for lipid and 50/50 PBd/PC hybrids are in between the values earlier reported by Paxton et al. using the PBd_37_‐PEO_22_ polymer^37^ and the permeability coefficients identified by Seneviratne and colleagues24a for the smaller PBd_22_‐PEO_14_ polymer. Their results showed higher permeability for 25 % PBd‐PEO hybrids compared to liposomes, but in contrast lower permeability for 50 % PBd‐PEO hybrids. This difference might arise by the usage of slightly different lipid (POPC) compared to the egg PC used here or by minor variances in vesicle preparation. In general, their permeability coefficients are one order of magnitude lower than those reported here. This could be explained by the fact that they didn't use valinomycin in their experiments.

To our best knowledge, permeability coefficients for PDMS/PC and PDMS/PBd hybrids have not been reported yet. Only the water permeability of PDMS GUVs under osmotic stress has been earlier investigated by Carlsen and colleagues.^43^ The calculated permeability coefficients indicate PDMS/PC hybrids high apparent permeability, which may be ascribed to proton transport through transient pores. A literature study of PDMS GUVs has shown that these vesicles under stress conditions might form transient pores, without losing its integrity. The permeability coefficient of mixed PDMS/PBd vesicle, without consideration of an initial pH jump is similar to the permeability coefficient of the other membrane compositions.

### Long‐term stability in different compartments

**Figure 8:**
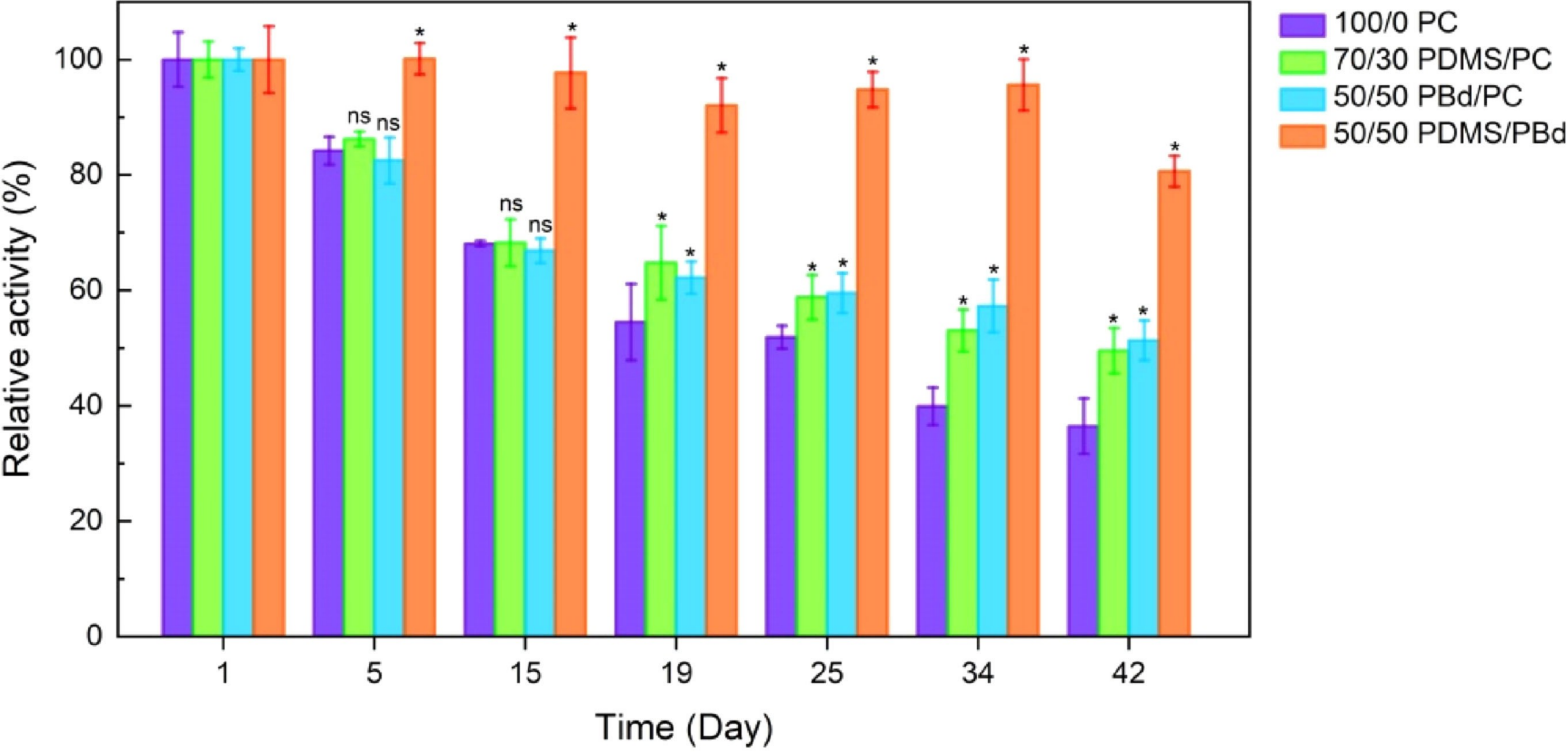
Hybrid vesicles improve long‐term stability of the ATP regeneration module. The activity is normalized to the activity at day 1. *P≤0.05, not significant (ns) *P* >0.05 (*P* values are generated by unequal variance t‐test (Welch's test) for comparison of each hybrid membrane composition to the lipid vesicle sample). *n*≥4; error bars represent standard error of the mean (SEM). Needs to be exchanged

According to the literature, one of the major benefits of hybrid and polymer vesicles over natural proteoliposomes is their enhanced functional durability.^13^ To prove this hypothesis, we monitored the activity of our system in different lipid/hybrid vesicles over 42 days (Figure 8).

Samples are stored at 4 °C during this period. To prove significant differences (*P≤0.05) between lipid and hybrid vesicles, we performed an unequal variance t‐test (Welch's test). Relative protein activities indicate the highest improvement of long‐term stability for 50/50 PDMS/PBd. Significant differences are evidenced from day 5 on with remaining activities of around 80 % after 42 days. For hybrid vesicles made of lipid and polymer (70/30 PDMS/PC and 50/50 PBd/PC) significant enhancement of activity compared to liposomes is demonstrated from day 19 on. In addition to this our results show that not only the relative activity (activity normalized to day 1), but also the absolute protein activity in hybrid vesicles overreaches that of liposomes after day 19 (Figure S12).

Both lipid/polymer hybrid vesicles seem to improve stability in a similar way, while the stability in polymer/polymer hybrid vesicles is remarkably higher. This outcome is in good agreement with recently reported extended functional lifetime of cytochrome bo_3_ oxidase in PBd‐PEO hybrid vesicles.^13^, 24a Enhanced functional durability of membrane proteins could especially play an important role when designing biosensors, drug delivery systems, nanoreactors, energy capture, storage devices or artificial cells.

## Discussion

In this study, the successful integration of two complex transmembrane proteins, bR and F_O_F_1_ ATP synthase, in polymer/lipid and polymer/polymer hybrid vesicles is demonstrated. ATP production rates indicate that both enzymes retain their functionality in the polymer‐based surrounding.

Using a Triton X‐100 mediated reconstitution, we could achieve high protein activity in lipid/polymer (98 % and 95 %) and polymer/polymer‐based hybrid vesicles (56 %). Choi et al.^12^ reported absolute ATP production rates of 120 nmol (mg ATP synthase)^−1^ min^−1[8]^ when reconstituting TF_O_F_1_ ATP synthase and bR patches in ABA triblock polymersomes. Absolute ATP production rates governed here are slightly higher (255±8 nmol (mg ATP synthase)^−1^ min^−1^ for 70/30 PDMS/PC, 240±5 for 50/50 PBd/PC and 140±8 for 50/50 PDMS/PBd).

To quantify the effect of the membrane environment on the activity of both proteins in detail, future work should address limiting factors for ATP production as well as stoichiometric and substrate effects that have impact on the kinetic parameters of the enzyme (*K*_m_ and *v*_max_).

Turbidity profiles are used to precisely determine the amount of detergent necessary for partial solubilization of lipid and hybrid vesicles. We choose the destabilization point at the onset of solubilization, even though optimal protein performance for Triton X‐100 mediated reconstitution has been earlier reported for the intermediate step of total solubilization.11g, 44 This has been done to account for additional detergent brought in the reconstitution mixture by Triton X‐100 solubilized bR, which was slightly higher as reported by Pitard et al.11g

DLS data after detergent treatment evidenced that all vesicles remain intact and are existent as detergent‐saturated membranes (onset of solubilization). In contrast, significant differences between lipid and hybrid vesicles are obtained after removal of detergent using bio beads. Liposomes remain their original size while all hybrid vesicles seem to split in two distinct fractions of different size (∼75 nm and 400–600 nm). Khan et al.^13^ showed comparable DLS data after reconstitution of bo_3_ oxidase in 50/50 PBd/PC and 75 PBd/PC. DLS data in their study also indicated two distinct fractions, but with comparably smaller molecular size (∼10 nm and ∼100 nm). Cryo‐TEM images of 50/50 PBd/PC in contrast evidenced large worm‐like micelles of several microns length coexisting with the vesicles. In general, DLS data from highly non‐spherical particles needs to be interpreted carefully and cryo‐TEM images should be taken in the future to investigate hybrid vesicle formation during reconstitution.

Interestingly, the splitting in two fractions upon detergent removal, seem to have no big impact on the global ATP production rates in the present case. It might be possible that even higher rates are attainable if these two fractions would be separated. Unlike lipid‐detergent interactions, detergent‐polymer interactions are currently not well understood24b and measurements are necessary to clarify differences between lipid and polymer. The molecular understanding of these interplays will enable adjustment of reconstitution procedures in an appropriate way.

In contrast to the relative high performance of the co‐reconstituted system in hybrid vesicles, the performance of bR in hybrid vesicles seem to be comparably low. The steady‐state ΔpH reached in 70/30 PDMS/PC and 50/50 PBd/PC remains only ∼30 % compared to liposomes. The steady‐state ΔpH of 50/50 PDMS/PBd is with ∼14 % even lower.

According to Seignereut et al.^29^ light‐induced proton uptake in bR liposomes is determined by three factors: the number of active pumps, the passive permeability of the membrane, and the back‐pressure effects (potential gradient ΔΨ and pH gradient ΔpH) that inhibits proton pumping.

Back‐pressure effects caused by the build‐up of charge (potential gradients ΔΨ ) are only relevant when the non‐proton permeability of the membrane is low. These effects are overcome in the present case by the addition of valinomycin. Back‐pressure effects caused by the proton gradient itself (concentration gradient ΔpH) are mainly influenced by the buffering capacity and should be constant for all membrane compositions. Therefore, discrepancies of ΔpH in different vesicles should be either explained by the number of active pumping units or by the passive proton permeability of the membrane.

The number of pumping units is determined by reconstitution efficiency and orientation of bR in the membrane. Slightly lower reconstitution efficiencies are detected in hybrid vesicles (71–78 %) compared to lipid vesicles (82 %) and the bR digestion assay strongly indicate that there is more bR correctly orientated in liposomes compared to hybrid vesicles. Anyway, the number of active bR molecules should only influence initial pumping rates. Steady‐state ΔpH values should be constant due to back‐pressure effects of ΔpH (Figure S6).

When the conditions are met in a way that the non‐proton permeability of the membrane is large (e. g., by addition of valinomycin), the establishment of steady‐state ΔpH is to a certain extent determined by the passive proton‐back leakage.^29^ Proton permeability determined here is higher for hybrid vesicles compared to liposomes and might explain comparably small steady‐state ΔpH in bR‐hybrid vesicles. In general, steady‐state ΔpH is reached when pumping and leakage rates are identical.

Even though steady‐state ΔpH values in hybrid vesicles are low, the ATP production rates in bR‐EF_O_F_1_ ATP synthase vesicles are barely influenced by this. We believe that in the case of the co‐reconstituted system, protons pumped by bR can efficiently diffuse along the membrane surface between the source (bR) and the sink (ATP synthase) without dissipation losses into the aqueous bulk as recently evidenced by Heberle et al.^45^ Besides, no back‐pressure effects are relevant in the co‐reconstituted system and the non‐proton permeability is low due to the absence of valinomycin. Under these conditions passive proton leakage has only little influence.

The here presented long‐term stability measurements clearly evidence the advantageous in using hybrid vesicles instead of liposomes. This enhanced stability may not only be critical to synthetic biology but could also become an important tool in handling membrane proteins in fundamental biochemical studies.24b Both lipid‐polymer hybrid vesicles seem to increase the long‐term stability in a similar way, while the activity at day 1 is slightly better in 70/30 PDMS/PC vesicles. We mainly attribute this to the higher fluidity of the PDMS polymer compared to PBd. PBd in contrast is packed more tightly in the membrane which might cause higher steric interactions between the hydrophilic PEO chains of the polymer and the head of the ATP synthase. Anyway, this increased viscosity compared to lipid membranes is also supposed to be a critical factor in stabilizing proteins over time.24a In general, the flexibility of the polymer chains and the hydrophobic thickness of the membrane are important parameters for successful integration of membrane proteins: flexible, linear hydrophobic polymers allow conformational adaption to the preferred hydrophobic thickness of the protein.24b

Jacobs and colleagues^15^ recently showed increased folding of a membrane protein during cell‐free expression when using PBd hybrid membranes instead of pure lipid membranes. In their work, they evidenced that the mechanical properties of the membrane (e. g., the area expansion module) highly influence the interactions between protein and membrane. Changes in membrane elastic properties can lower or increase the energy of membrane deformation and can therefore increase or decrease the conformational freedom of a protein. The decrease of conformational freedom might slow down the process of protein unfolding and therefore increase long‐term stability.

Further optimization of membrane composition (e. g., different polymers and/or lipids) and reconstitution procedure, might further increase biocompatibility and long‐term stability. The lipid‐polymer or polymer‐polymer ratio seems to be another important parameter influencing the chemical and mechanical properties of the membrane and therefore the interaction with proteins embedded in them. Phase‐separated membranes with lipid‐ and polymer‐rich domains might be also attractive for transmembrane reconstitution, combining native‐like lipid solvation with the enhanced structural stability of polymersomes.

## Conclusion

In the present work a detailed study of membrane protein co‐reconstitution in hybrid membranes based on two different polymers PBd_22_‐PEO_14_ and PDMS‐g‐PEO with different hydrophobic blocks as well as varying architecture is performed. We demonstrate a co‐reconstitution procedure for complex membrane proteins into hybrid vesicles with high remaining performance (98 % in PDMS‐g‐PEO hybrids and 92 % in PBd‐PEO hybrids). Moreover, a significant enhancement of protein long‐term stability could be proven in lipid/polymer‐based vesicles as well as in polymer/polymer‐based vesicles.

## Experimental Section

**Materials**: Soy L‐α‐phosphatidylcholine (PC, 95 %) was purchased from Avanti Polar Lipids. Polymer PDMS‐g‐PEO was a kind gift from Dow Corning. The polymer had an average viscous‐metric molecular weight of 3000 g/mol with 47 % weight fraction of ethylene oxide (in average 2 arms of PEO per PDMS chain) and an average degree of polymerization of 12. Poly(butadiene‐b‐ethylene oxide) (PBd_22_‐b‐PEO_14_) was purchased from Polymer Source (P9089‐BdEO) with an average molecular weight of 1200 for the PB and 600 for the PEO block. SM‐2 Bio‐Beads derived from Bio‐Rad were extensively washed before usage as described by Holloway.^46^ Luciferin/Luciferase reagent CLSII from Roche was prepared as a 10 times concentrated solution. Ultra‐pure ADP was purchased from Cell Technology. All other reagents were obtained from Sigma Aldrich.

**Expression and purification of membrane proteins**: Purple membrane was isolated from *Halobacterium salinarium* (strain S9) as described by Oesterhelt and Stoeckenius.20b His‐tagged *E. coli* F_O_F_1_‐ATP synthase (EF_O_F_1_) was expressed from the plasmid pBWU13‐βHis in the *E. coli* strain DK8 (ΔuncBEFHAGDC) and purified by Ni‐NTA affinity chromatography as previously described by Ishmukhametov.20a

**Preparation of lipid and hybrid vesicles for light‐driven ATP production**: Vesicles were formed by film rehydration method followed by extrusion. 10 mg of dissolved lipid/polymer was deposited in a glass vial and solvent was removed using a gentle stream of nitrogen. PDMS‐g‐PEO/PC were mixed 70/30 (m/m), PBd‐PEO/PC were mixed 50/50 (m/m) and PDMS‐g‐PEO/PBd‐PEO were mixed 50/50 (m/m). Thin lipid/polymer films were rehydrated in vesicle buffer (20 mM HEPES (pH 7.5), 2.5 mM MgSO_4_, 50 mg/mL sucrose) to a final concentration of 10 mg/mL by vortexing. To transform multilamellar vesicles into unilamellar vesicles, the suspension was subjected to 5 freeze‐thaw cycles. Each cycle consisted of freezing in liquid nitrogen, thawing in a 35 °C water bath and vortexing for 30 s. For PBd‐PEO hybrid vesicles the thawing temperature was 60 °C according to Seneviratne et al.24a Suspensions were extruded 11 times through a 100 nm pore size polycarbonate membrane (Whatman) to form uniform nanovesicles.

**Vesicle size and dispersity by dynamic light scattering**: The average vesicle size and dispersity were determined by dynamic light scattering (DLS) using a Zetasizer Nano ZS (Malvern, Worcestershire, UK) with a 633 nm helium‐neon laser and back‐scattering detection. 5 μL of vesicles were diluted in 1 mL vesicle buffer and samples were measured at a fixed 173° scattering angle at 25 °C. All reported values are based on the average of three measurements. Each measurement consisted of 3×5 runs with 70s duration.

**Solubilization of bR patches**: bR patches were solubilized according to the method described by Meyer et al.^47^ bR patches were supplemented with Triton X‐100 (Triton) in a Triton to bR molar ratio of 68. The suspension was sonicated for ∼10 min in an ultrasonic bath and stirred for 4 days in the dark at 4 °C. Membrane pellets were removed by ultracentrifugation for 30 min at 400 000 *g*.

**Co‐reconstitution of EF_O_F_1_‐ATP synthase and bR**: 100 μL of preformed lipid and hybrid vesicles were mixed with 0.1 μM EF_O_F_1_‐ATP synthase and 2.9 μM bR as monomeric protein in detergent. To solubilize the vesicles partially, 0.3 % Triton (100/0 PC) or 0.06 % Triton (70/30 PDMS/PC, 50/50 PBd/PC and 50/50 PDMS/PBd) were added under vortexing. After 15 min incubation in the dark under gentle shaking, 30 mg (100/0 PC) or 6 mg (70/30 PDMS/PC, 50/50 PBd/PC and 50/50 PDMS/PBd) wet SM‐2 Bio‐Beads were added and the solution was incubated for further 60 minutes under constant shaking in the dark.

**Light‐induced ATP production** : For measurement of light‐induced ATP production, 25 μL of co‐reconstituted vesicles were diluted in 250 μL measurement buffer (10 mM Tris‐HCl (pH 7.5), 50 mM KCl, 2 mM MgCl_2_, 5 mM potassium phosphate, 1 mM DTT) containing 10 μL ADP (7.9 mM). The reaction was started by illumination with a 50 W green LED lamp (SMD RGB Floodlight, V‐TAC). Aliquots of 25 μL were taken every 5 minutes from the reaction mixtures and the reaction was stopped by addition of the same volume of trichloroacetic acid (40 g/L). The ATP concentration was measured with the luciferin/luciferase assay and calibrated by addition of 10 μL ATP (7.8 μM) after each measurement. Calculation of ATP is shown in the Supporting Information.

**Preparation of lipid and hybrid vesicles for bR proton pumping**: Vesicles were prepared as described above with slight modifications. Thin lipid/polymer films were rehydrated in HEPES buffer (10 mM HEPES (pH 7.0), 100 mM K_2_SO_4_, 15 mM MgCl_2_) in the presence of 10 mM 8‐hydroxyprene‐1,3,6‐trisulfonic acid (pyranine) to final concentration of 10 mg/mL.

**Reconstitution of bR and determination of reconstitution efficiency**: 100 μL of preformed lipid and hybrid vesicles were mixed with 80 μL of 60 μg/mL bR as monomeric protein in detergent. To solubilize the vesicles partially, 0.3 % Triton (100/0 PC) or 0.06 % Triton (70/30 PDMS/PC, 50/50 PBd/PC and 50/50 PDMS/PBd) were added under vortexing. After 15 min incubation in the dark under gentle shaking, 30 mg (100/0 PC) or 6 mg (70/30 PDMS/PC, 50/50 PBd/PC and 50/50 PDMS/PBd) wet SM‐2 Bio‐Beads were added and the solution was incubated for further 60 min under constant shaking in the dark.

For the removal of non‐encapsulated pyranine, vesicles were loaded on a pre‐packed G25 size exclusion column (PD Mini Trap^TM^ G‐25, GE Healthcare). The reconstitution efficiency was calculated from the absorbance ratio at 560 nm before and after gel filtration.

**bR proton pumping activity**: The proton pumping activity of bR was monitored using the pH sensitive dye pyranine. Before each measurement, 0.1 μM valinomycin was added to the solution to avoid the formation of a potential gradient that counteracts the generated pH gradient. After 1 h of equilibration in the dark, the reaction was started by illumination with a 50 W green LED lamp (SMD RGB Floodlight, V‐TAC). The absorption change of pyranine at 405 and 450 nm was monitored using a diode array spectrometer (QEPRO, Ocean Optics). The pH was calculated by the absorbance ratio between 450 and 405 nm (*A*_450_/*A*_405_) using a calibration curve as described by Seneviratne et al.24a Each proton pumping rate was the average of at least three independent measurements.

**bR orientation by proteolytic cleavage**: Determination of bR orientation by proteolytic cleavage was performed according to Gerber et al.^31^ To assay the orientation of bR in lipid and hybrid vesicles, proteinase K (Roche) was added to a final concentration of 2.5 mg/mL. After incubation for 2 hours at 37 °C, the reaction was stopped by adding the protease inhibitor phenylmethanesulfonylfluoride to a concentration of 10 mM while cooling the reaction on ice for 30 minutes. The reaction products were loaded onto 4–20 % Tris‐HCl Criterion Precast Gels (Bio‐Rad).

**Proton permeability measurements**: Vesicles were prepared as described in preparation of lipid, hybrid and polymer vesicles with slight modification. After evaporation, lipid/polymer were rehydrated in PIPES buffer (25 mM PIPES, 200 mM sucrose, pH 7.5) adjusted to pH 7.5 with KOH (50 mM) in the presence of 10 mM pyranine. Vesicles were subjected to 5 freeze‐thaw cycles, extruded through 100 nm pores and loaded on a pre‐packed G25 size exclusion column (PD Mini Trap^TM^ G‐25, GE Healthcare) to remove not encapsulated pyranine. For permeability measurements 34 μL vesicles were diluted in 800 μL PIPES buffer supplemented with 180 nM valinomycin (or without valinomycin) and the reaction was started by addition of 20 μL 100 mM HCl/ 13 μL 100 mM NaOH. The absorption change of pyranine at 405 and 450 nm was monitored over 1 h using a diode array spectrometer as described above.

**Supporting Information**: Calculation of ATP concentration using the luminescence signal, SDS‐PAGE of purified proteins, ATP production with different bR concentration, Triton X‐100 destabilization profiles for different lipid and hybrid vesicles, pH determination with pyranine, Proton pumping rates depending on the number of pumping units, SDS‐PAGE analysis of reconstituted bR patches, Permeability measurements, Determination of Permeability coefficient P, p‐values as determined by Welch's test for long‐term stability, Absolute protein stability in different compartments over time

## Conflict of interest

The authors declare no conflict of interest.

## Supporting information

As a service to our authors and readers, this journal provides supporting information supplied by the authors. Such materials are peer reviewed and may be re‐organized for online delivery, but are not copy‐edited or typeset. Technical support issues arising from supporting information (other than missing files) should be addressed to the authors.

Supplementary
