# Supplementary material for: Light‐Driven ATP Regeneration in Diblock/Grafted Hybrid Vesicles
Source: Chembiochem. 2020 Apr 7;21(15):2149–60. doi: 10.1002/cbic.201900774 (PMC7496644; doi:10.1002/cbic.201900774)
Supplement: Supplementary file 1 — Supplementary [file CBIC-21-2149-s001.pdf]

ChemBioChem

Supporting Information

# Light-Driven ATP Regeneration in Diblock/Grafted Hybrid Vesicles

Christin Kleineberg, Christian Wölfer, Amirhossein Abbasnia, Dennis Pischel,
Claudia Bednarz, Ivan Ivanov, Thomas Heitkamp, Michael Börsch, Kai Sundmacher, and
Tanja Vidaković-Koch*© 2020 The Authors. Published by Wiley-VCH Verlag GmbH & Co.
KGaA. This is an open access article under the terms of the Creative Commons Attribution
License, which permits use, distribution and reproduction in any medium, provided the
original work is properly cited.

## Calculation of ATP concentration using the luminescence signal

$$c_{syn} = \frac{I_S - (I_B \cdot \frac{V_0}{V_1})}{I_C - (I_S \cdot \frac{V_1}{V_2})} \cdot \frac{C_C \cdot V_C}{V_2} \cdot \frac{V_1}{V_S} \cdot \frac{V_{TCA}}{V_{syn}}$$

$c_{syn}$: Concentration of ATP during synthesis

$I_B$: Background luminescence Intensity

$I_S$: Sample luminescence Intensity

$I_C$: Calibration luminescence Intensity

$V_0$: Volume of luciferase [200 µl]

$V_1$: Volume of luciferase + Sample [220 µl]

$V_2$: Volume of luciferase + sample + calibration [230 µl]

$C_C$: Concentration of ATP in Calibration [7800 nM]

$V_C$: Volume of Calibration [10 µl]

$V_S$: Volume of Sample [20 µl]

$V_{syn}$: Volume of sample when stopping the reaction [25 µl]

$V_{TCA}$: Volume of sample when stopping the reaction + Volume of TCA [50 µl]

## SDS-PAGE of purified proteins

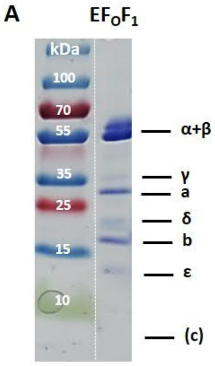


| Subunit | Molecular mass (kDa) |
|---|---|
| α | 55.3 |
| β | 50.3 |
| γ | 31.6 |
| δ | 19.3 |
| ε | 14.9 |
| a | 30.3 |
| b | 17.2 |
| c | 8.3 |

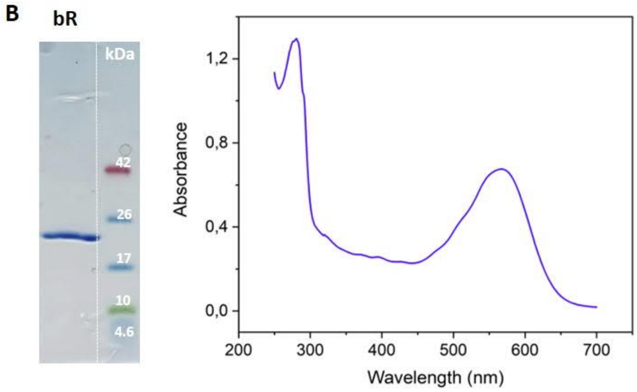


Figure S1. SDS-PAGE of purified proteins. (A) Coomassie-stained SDS-PAGE of purified $EF_OF_1$ ATP synthase and molecular masses of $EF_OF_1$ subunits. (B) Coomassie-stained SDS-PAGE of purified bR with the corresponding absorbance spectrum.

## ATP production with different bR concentration

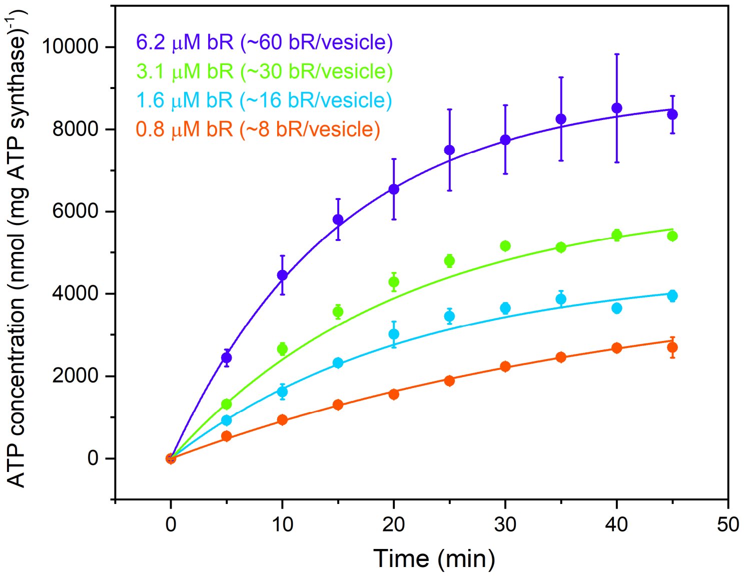


Figure S2. ATP production after co-reconstitution with 1 $EF_OF_1$/liposomes and various amounts of bR. The maximal concentration of bR is governed by the stock bR concentration from the isolation protocol. bR is reconstituted in form of patches to avoid material loss during solubilization.

<u>Triton X-100 destabilization profiles for different lipid and hybrid vesicles</u>

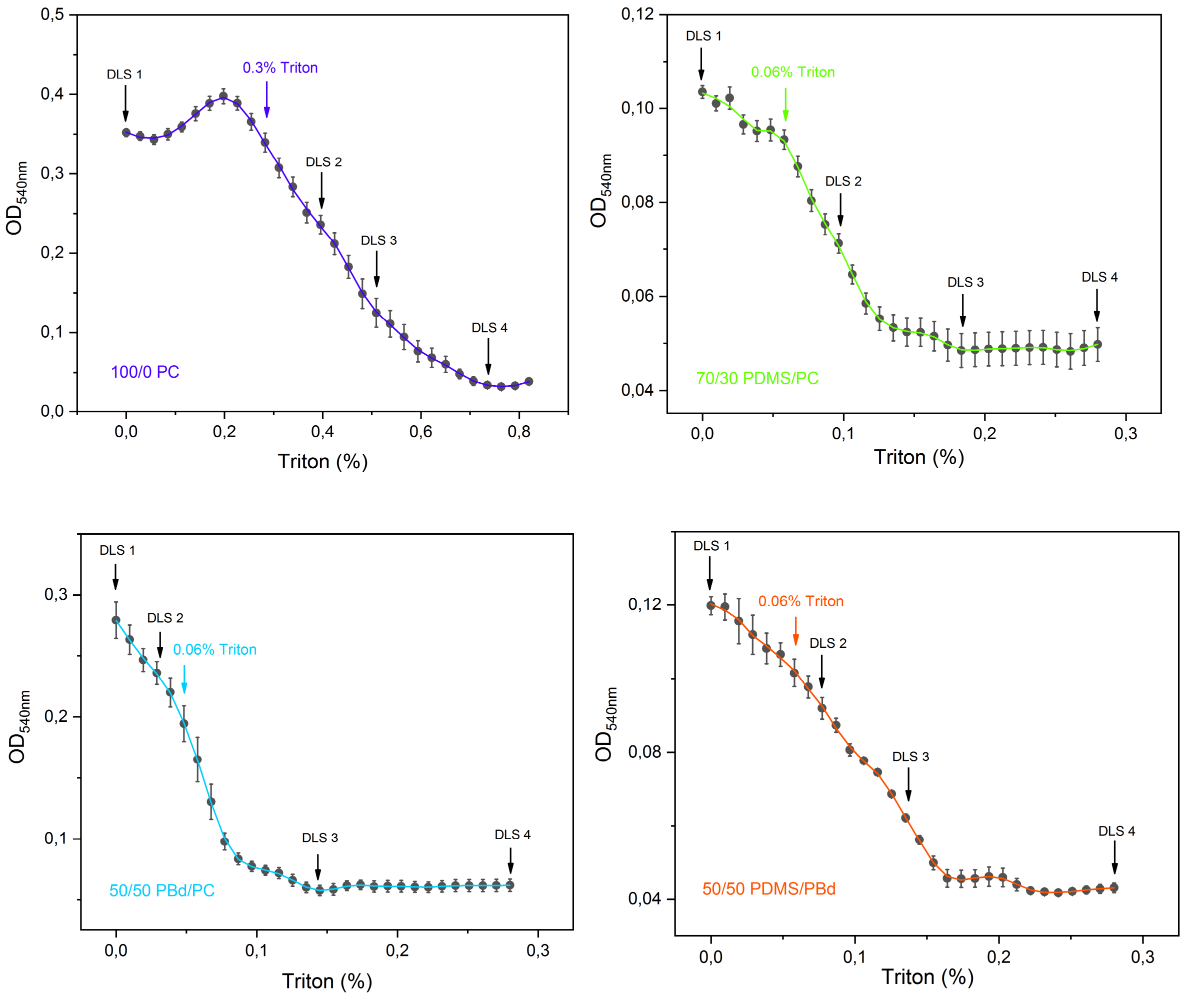


Figure S3. Detergent destabilization profiles for vesicles made of 100/0 PC, 70/30 PDMS/PC, 50/50 PBd/PC and 50/50 PDMS/PBd. Plots show the change in absorption at 540 nm upon addition of several amounts of Triton X-100. The destabilization point with the corresponding amount of Triton X-100 chosen for reconstitution is indicated with a colorful arrow. DLS measurements have been performed at several points of destabilization marked as DLS1-4 and are shown in Figure S4.

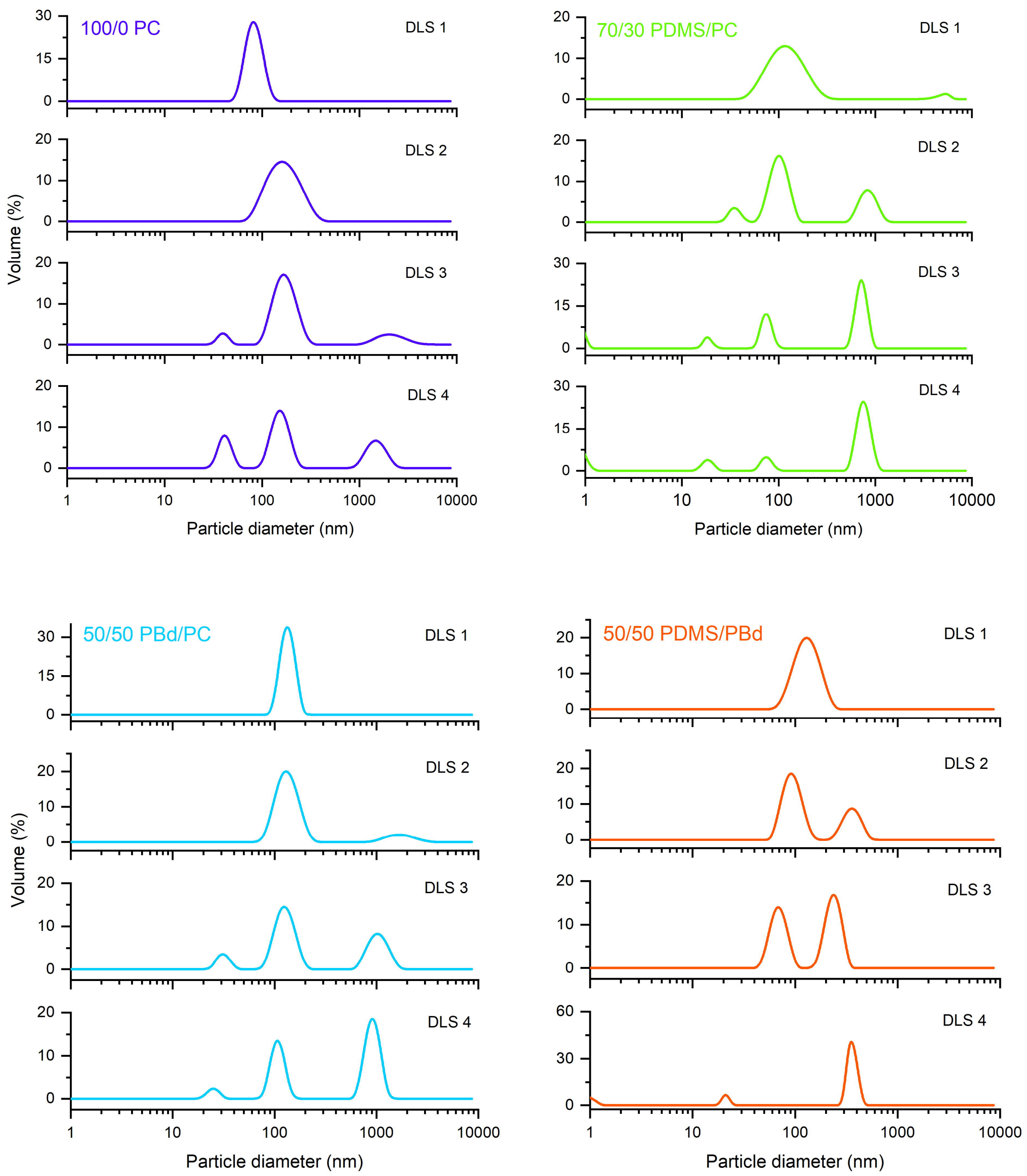


Figure S4. Size distribution of hybrid vesicles made of 100/0 PC, 70/30 PDMS/PC, 50/50 PBd/PC and 50/50 PDMS/PBd. Plots show vesicle size distribution at different points of destabilization (DLS 1-4 in Figure S3).

## pH determination with pyranine

For determination of the internal pH of vesicles a standard curve (Figure S5) is generated. The pH of a continuously stirred 1 mM pyranine solution in 40 mM HEPES buffer is monitored with a pH electrode and adjusted by addition of small amounts of 1 M KOH. At regular pH intervals (~0.2) 500 µl of the solution is taken and analyzed by measurement of absorption at 405 nm and 450 nm.

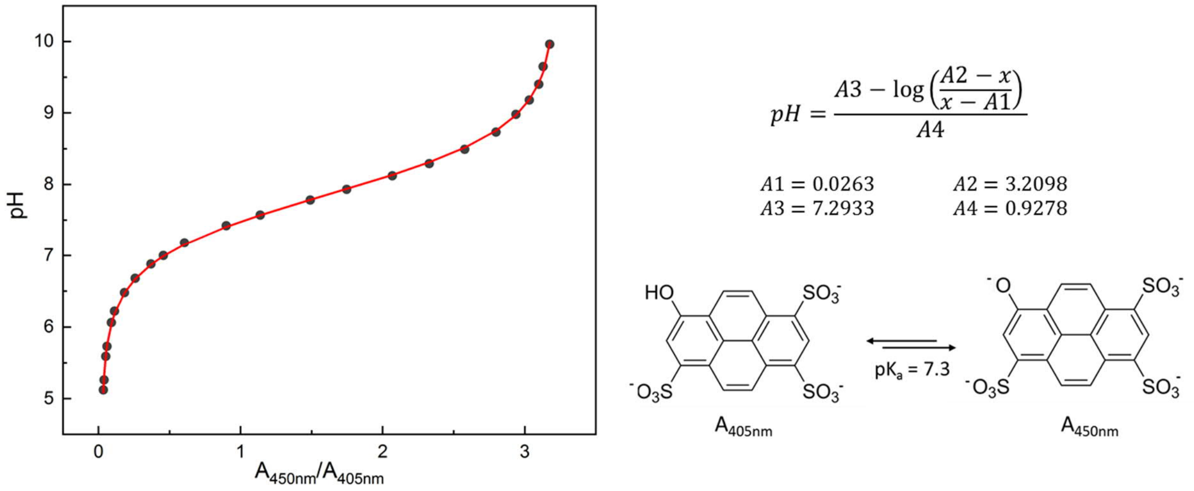


Figure S5. Calibration curve for determination of pH by pyranine absorption at 450 nm ($A_{450nm}$) and 405 nm ($A_{405nm}$). The measurement points are fitted to a formulation of the Henderson-Hasselbach relation (upper right side, equation S1) using Matlab.

## Proton pumping rates depending on the number of pumping units

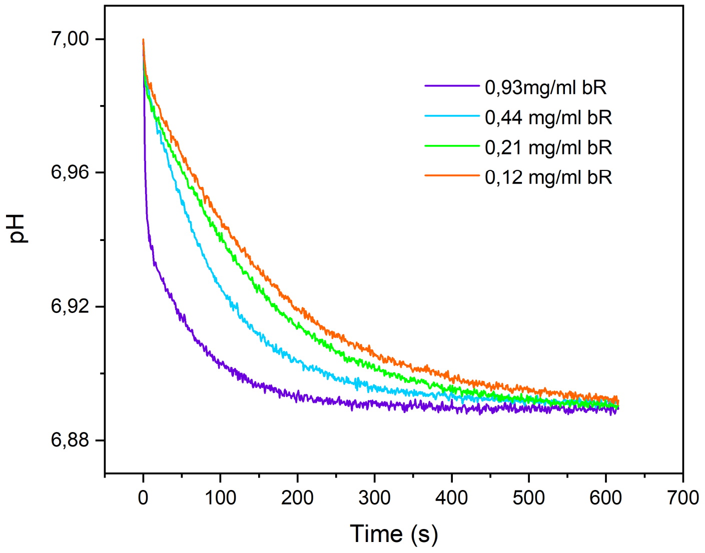


Figure S6. Effect of the lipid to protein ratio on bR proton pumping rates. Initial rates of proton pumping increase with protein content, while the steady state ΔpH remains unchanged.

## SDS PAGE analysis of reconstituted bR patches

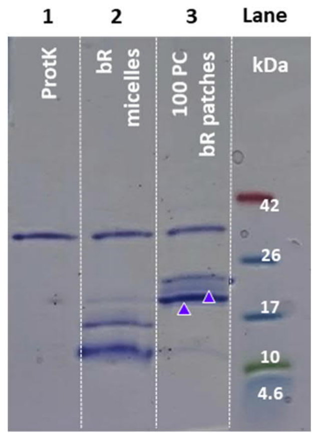


Figure S7. SDS PAGE after proteolytic cleavage of reconstituted bR with proteinase K (ProtK) shows uniform orientation of bR with the C-terminus facing outwards. Lane 1: band specific for ProtK enzyme only; lane 2: digestion product of not reconstituted bR; lane 3: digestion product of bR patches reconstituted in lipid vesicles.

## Permeability measurements

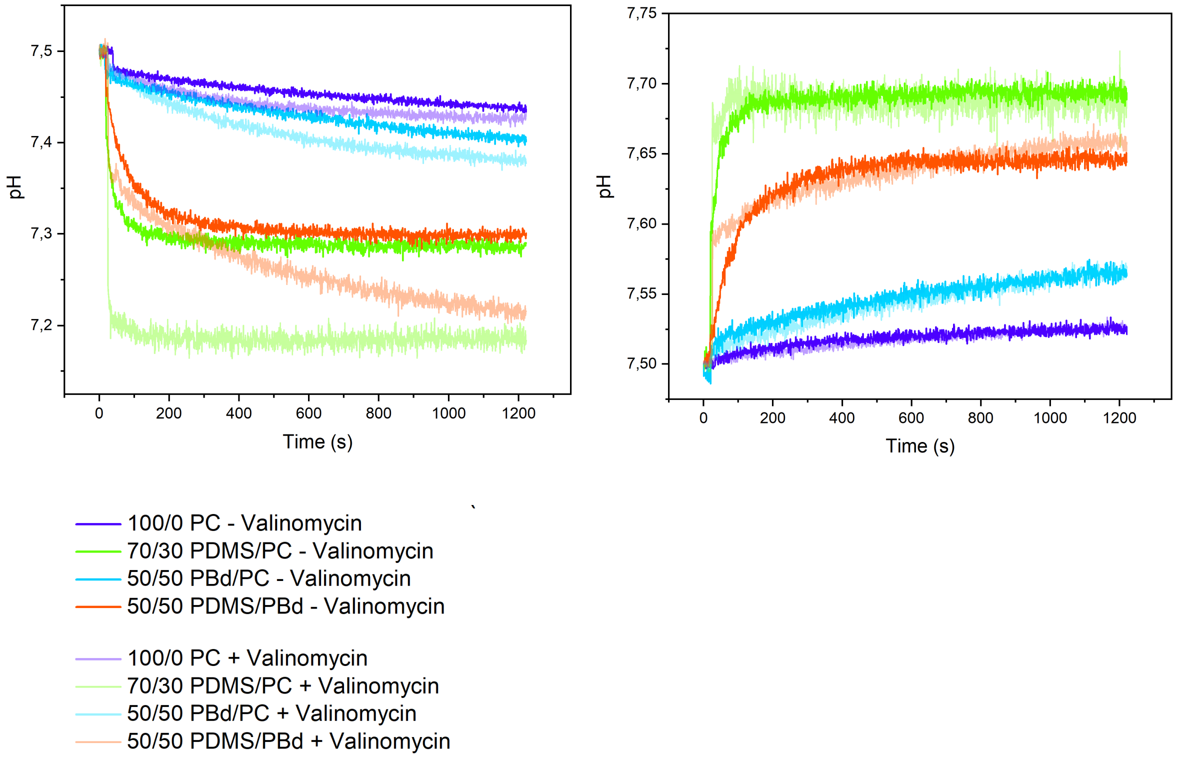


Figure S8. Proton permeability of vesicles as measured by pH change after addition of 2.4 mM HCl (left) and 1.6 mM NaOH (right) to the outer solution. Proton permeability in the presence of valinomycin (+ Valinomycin) is compared to proton permeability in the absence of valinomycin (- Valinomycin).

Determination of Permeability coefficient P

## 1) Determination of P by Model fitting (Method 1)

Since the buffering properties of the system influence the pH change in addition to membrane permeability (see Fig. S9), the dynamic buffering capacity β should be taken into account when determining the permeability constant P independent of the buffer system. The buffering capacity is defined as the proton concentration to be added to achieve a pH change:

$$\beta = -\frac{d[H^+]}{dpH} \qquad [S2]$$

Therefore, the pH rate applies taking into account the chain rule:

$$\frac{dpH}{dt} = \frac{dpH}{d[H^+]} \cdot \frac{d[H^+]}{dt} \qquad [S3]$$

$$\frac{dpH}{dt} = -\beta \cdot \frac{d[H^+]}{dt} \qquad [S4]$$

Assuming that the pH value inside the vesicle changes only due to proton permeability through the membrane ($r_L$), the following rate for the pH value inside the vesicle results, considering the total volume ratio between inner and exterior phase $V_R = \frac{V_I}{V_E}$:

$$\frac{dpH_I}{dt} = \frac{-\beta}{V_R} \cdot (-r_L) \qquad [S5]$$

Due to the vesicle concentration of $7{\cdot}10^{12}$ liposomes/mL with a mean diameter of 125 nm the ratio of bulk vesicle volume to external volume is very small ($V_R = 0.009$). Since the pH value in the outer and inner medium is also buffered (here PIPES; $pK_B = 6.7$), the external proton change is very small and therefore negligible. In the following, the equation for dynamic buffer capacity β is derived by buffer equilibrium:

$$B^- + H^+ \leftrightarrow BH \qquad [S6]$$

By considering the law of mass action and the buffer equilibrium constant $K_b$ follows:

$$[BH] = \frac{[H^+][B^-]}{K_b} \qquad [S7]$$

The total buffer concentration $c_b$ is defined as:

$$c_b = B^- + BH \qquad [S8]$$

Combining equation [S7] and [S8] it follows:

$$[B^-] = \frac{c_b \cdot K_b}{K_b + [H^+]}$$ [S9]

Taking the charge balance

$$[B^-] + [OH^-] = [H^+] + [n]$$ [S10]

into account, the following applies:

$$[n] = \frac{K_w}{[H^+]} - [H^+] + \frac{c_b \cdot K_b}{K_b + [H^+]}$$ [S11]

By calculating the derivatives, the equation for dynamic buffer capacity follows:

$$\beta = \frac{dn}{dpH} = \frac{dn}{dH^+} \cdot \frac{dH^+}{dpH}$$ [S12]

$$= \left[-\frac{K_w}{[H^+]^2} - 1 - \frac{c_b \cdot K_b}{(k_b + [H^+])^2}\right] \cdot (-\ln(10) \cdot [H^+])$$ [S13]

$$= \ln(10) \cdot \left[\frac{K_w}{[H^+]} + [H^+] + \frac{c_b \cdot K_b \cdot [H^+]}{(K_b + [H^+])^2}\right]$$ [S14]

Equation [S14] shows that at $pK_B$ (derived from the buffer constant) the buffer system has the largest buffer capacity (Fig. S9). For pH values close to $pK_B$, protons must be added to the inner medium the most in order to obtain a pH change.

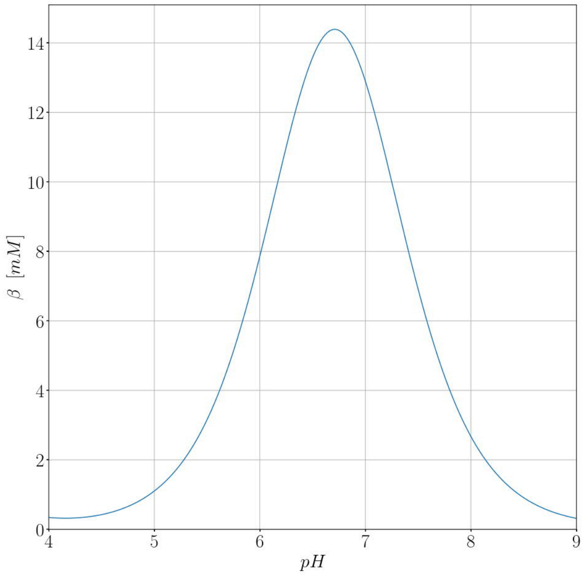


Figure S9. Buffer capacity (eq. S14) for different pH (Pipes buffer: $pK_B$ = 6.71, $c_B$ = 25 mM).

The experimental observations of proton permeability showed that pH change inside the vesicles upon the addition of acid/base never tends towards the external pH in equilibrium. For NaOH the pH inside is always below the external pH and for HCl always above the external pH. This indicates that in the experiment the membrane permeability of protons is not only driven by the chemical gradient but also by the membrane potential built up by different internal and external ion concentrations. For this reason, a permeability rate $r_L$ is derived using the Nernst-Planck equation, which takes both the chemical and electrical potential ($E_m$) into account:

$$j = -D_c \left( \frac{dc}{dx} - \frac{z \cdot F}{R \cdot T} \cdot \frac{E_m}{L} \cdot c \right) \qquad \text{[S15]}$$

The terms of the Nernst-Planck equation represent the Fick's law of diffusion, which gives the flux due to diffusion and the flux due to the electric potential. Using separation of variables and integrate between interior (x=0) and exterior (x=L) yields to:

$$j = P_c \cdot \mu \cdot E_m \cdot \left( \frac{c_e - c_i \cdot e^{\mu E_m}}{1 - e^{\mu E_m}} \right) \qquad \text{[S16]}$$

with

$$\mu = -\frac{F}{R \cdot T} \qquad \text{[S17]}$$

And the permeability coefficient $P_c$

$$P_c = \frac{D_c}{L} \qquad \text{[S17]}$$

Assuming spherical vesicles leads to the surface volume ratio:

$$\frac{A}{V} = \frac{3}{\rho} \qquad \text{[S18]}$$

Combining equations [S16]-[S18] the permeability rate $r_L$ can be defined as follows:

$$r_L = P_H \cdot \mu \cdot \frac{3E_m}{\rho} \cdot \left( \frac{[H_e^+] - [H_i^+] \cdot e^{\mu E_m}}{1 - e^{\mu E_m}} \right) \qquad \text{[S19]}$$

Equation [S19] only applies to constant electrical potential. Since in the present case the membrane potential is mainly determined by the ion imbalance between the inner and outer phase in the individual experiments ($Na^+$ or $Cl^-$), which can only balance very slowly through the membrane, it is assumed that the membrane potential is approximately constant over the observed period. With vesicle radius ρ = 125 nm as determined by DLS. If no membrane potential is present, the kinetic is simplified by applying the rule of L'Hopital to the purely chemical driven rate:

$$r_L = P_H \cdot \frac{3}{\rho} \cdot ([H_e^+] - [H_i^+]) \qquad [S20]$$

This correlation is used by Paxton et al. [1] for the determination of permeability coefficients and is described below.

Parameter sets were estimated (Table S1) to fit the experimental datasets, consisting of a permeability constant per membrane type with a membrane potential for acid and base addition to the external medium, respectively. The parameter estimation was performed by minimizing the residual sum of squares RSS between simulation and experimental datasets, using the toolbox Copasi[2]. The optimization algorithm evolutionary programming was used to identify an approximation of a parameter set for a suitable global minimum of the RSS[3]. Additionally, the gradient orientated simplex algorithm was applied to certainly reduce the RSS into potential global minimum and optimal parameter set, respectively[4].

Table S1. Fitted parameter set.

| Membrane composition | | $P_H \times 10^9$ (cms$^{-1}$) | $E_m$ (mV) $pH_e$ = 7.77 | $E_m$ (mV) $pH_e$ = 7.11 |
|---|---|---|---|---|
| 100/0 | PC | 1.88 | 13.7 | -18.0 |
| 70/30 | PDMS/PC | 1100 | 5.0 | -4.6 |
| 50/50 | PBd/PC | 2.41 | 9.9 | -14.5 |
| 50/50 | PDMS/PBd | 2.96 | 5.7 | -5.3 |

The calculated membrane potentials correspond to the expected charge difference after acid or base addition. The addition of acid to the external medium leads to an excess of negative ions and thus to a negative membrane potential. The opposite counts for the addition of base (Table S1).

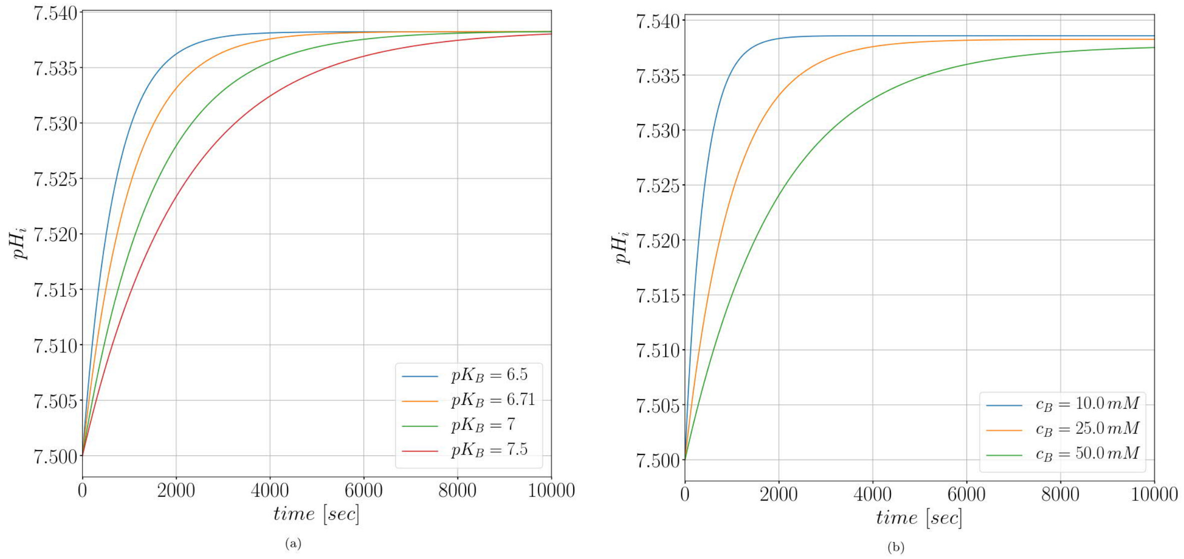


Figure S10. pH change inside of vesicles depending on different (a) $pK_B$ and (b) buffer concentrations (Lipsome: initial pH = 7.5, external pH = 7.77).

## 2) Determination of P according to Paxton et al. [1] (Method 2)

Permeability coefficients (P) are calculated using a method recently reported by Paxton et al.[1] The pH is determined using the pyranine standard curve shown in Figure S4. The pH values at t=0 are corrected to the pH of the buffer solution (pH=7.5). Some membrane systems show deviations between the actual and apparent pH due to interactions between pyranine and the vesicle material. As the pH for these experiments is in the linear range of the calibration curve, all data points could be offset by the same value for each series. The concentration of free hydroxide $[OH^-]$ or free protons $[H^+]$ could be calculated from the pH values using equation [S21] or [S22]:

$$[OH^-] = 10^{-pOH} = 10^{-(14-pH)} \qquad [S21]$$

$$[H^+] = 10^{-pH} \qquad [S22]$$

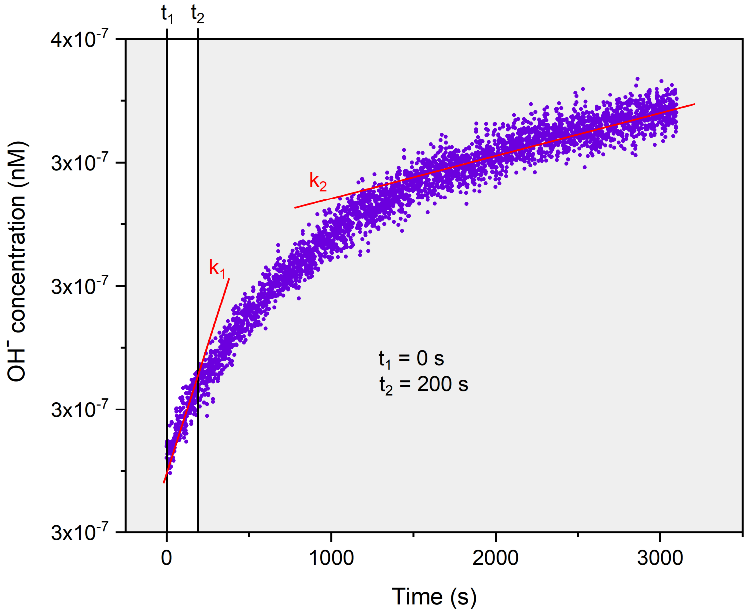


Figure S11. Determination of permeability coefficients (P) schematically shown for 100/0 PC lipids after addition of NaOH. The concentration of free [$OH^-$] is calculated from the pH response versus time by equation [S22]. The [$OH^-$] values at $t_1$ = 0s and $t_2$ = 200s are used for calculation of P.

For calculation of permeability coefficients the initial part of the reaction (0-200 s) has been chosen according to Seneviratne[5]. Proton permeation into vesicles occurs via a two-step mechanism [6] (shown as $k_1$ and $k_2$ in Figure S11). $k_1$ can be attributed to the rapid net $H^+$/$OH^-$ permeability[7] which results in an uncompensated build-up of charge[6c, 7]. The net $H^+$/$OH^-$ flux then slows to a rate, limited by the permeation of the charge-compensating co-ions ($k_2$). As $k_1$ actually reflects $H^+$/$OH^-$ permeability, this initial linear range of the reaction has been chosen for determination of P.

Calculation of the permeability coefficient $P_{OH-}$ is demonstrated for addition of NaOH. The permeability coefficient P is defined by the flux $r_L$ and the hydroxide ion concentration gradient Δc as follows:

$$P = \frac{r_L}{\Delta c} \qquad [S23]$$

$$\Delta c = [OH_i^-]_2 - [OH_i^-]_1 \qquad [S24]$$

The flux of hydroxide ions $r_L$ can be calculated by subtracting the flux $r_{L,2}$ at $t_2$ from the flux $r_{L,1}$ at $t_1$:

$$r_L = r_{L,2} - r_{L,1} \qquad [S25]$$

Whereby the flux $r_{L,1}$ and $r_{L,2}$ are:

$$r_{L,1} = \frac{[OH_i^-]_{t_1} + [PIPES^-]_{t_2} - [PIPES^-]_{t_1}}{\Delta t} \cdot \frac{V}{A} \qquad [S26]$$

$$r_{L,2} = \frac{[OH_i^-]_{t_2} + [PIPES^-]_{t_1} - [PIPES^-]_{t_2}}{\Delta t} \cdot \frac{V}{A} \qquad [S27]$$

V is the volume and A the surface area of the vesicle and can be calculated using the size distribution of vesicles determined by DLS:

$$V = \frac{4}{3} \cdot \pi \cdot \rho^3 \qquad [S28]$$

$$A = 4 \cdot \pi \cdot \rho^2 \qquad [S29]$$

The ionized form of PIPES $[PIPES^-]t_1$ and $[PIPES^-]t_2$ can be derived by using the $K_b$ value of PIPES with the following equations:

$$[PIPES^-]_{t_1} = \frac{[OH_i^-]_{t_1} \cdot [PIPES]}{K_b} = \frac{[OH_i^-]_{t_1} \cdot [PIPES]}{-0.86M} \qquad [S30]$$

$$[PIPES^-]_{t_2} = \frac{[OH_i^-]_{t_2} \cdot [PIPES]}{K_b} = \frac{[OH_i^-]_{t_2} \cdot [PIPES]}{-0.86M} \qquad [S31]$$

## p-values as determined by Welch's test for long-term stability

Table S2. Results for p-values as determined by unequal variance t-test (Welch's test) to prove significant enhancement of in hybrid vesicles compared to lipid vesicles. P-values < 0.05 indicate significant difference and are highlighted in red.

| **Membrane composition** | | Day 5 | Day 15 | Day 19 | Day 25 | Day 34 | Day 42 |
|---|---|---|---|---|---|---|---|
| 70/30 | PDMS/PC | 0,107 | 0,922 | 0,045 | 6,4E-3 | 6,5E-5 | 3,2E-3 |
| 50/50 | PBd/PC | 0,483 | 0,229 | 0,038 | 0,015 | 1,8E-4 | 1,9E-3 |
| 50/50 | PDMS/PBd | 8,4E-5 | 2,3E-3 | 6,8E-6 | 2,1E-6 | 3,2E-6 | 1,1E-5 |

Absolute protein stability in different compartments over time

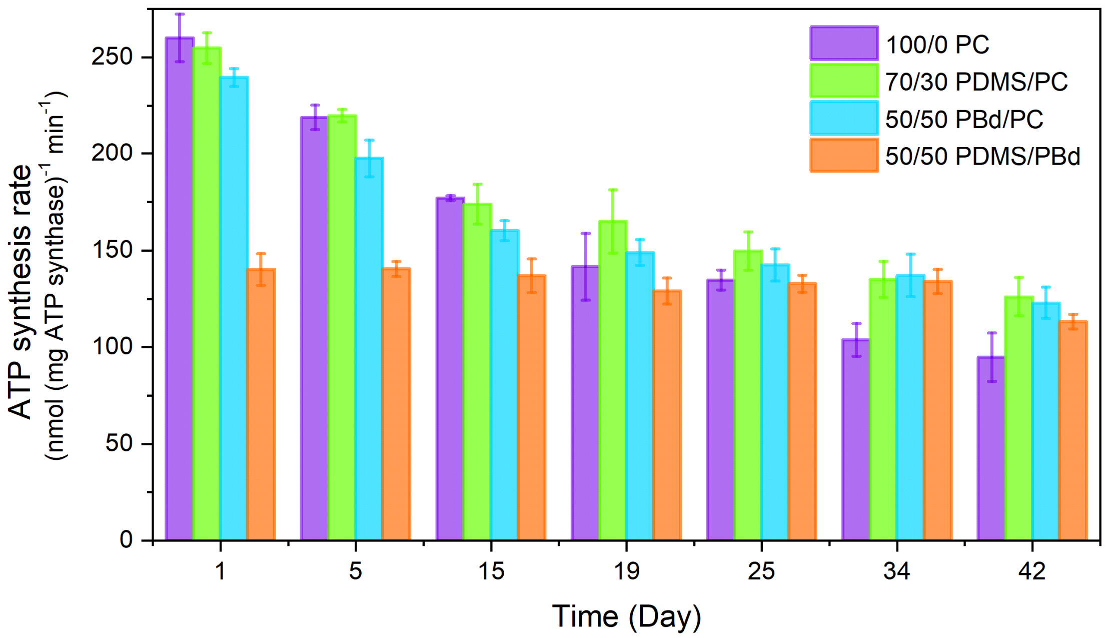


Figure S12. ATP synthesis rates over time in lipid and hybrid vesicles. After 19 days absolute ATP synthesis rates in hybrid vesicles overcome rates achieved in pure liposomes.

## REFERENCES


[1] W. F. Paxton, P. T. McAninch, K. E. Achyuthan, S. H. R. Shin, H. L. Monteith, Colloids and Surfaces B: Biointerfaces 2017, 159, 268-276.

[2] S. Hoops, R. Gauges, C. Lee, J. Pahle, N. Simus, M. Singhal, L. Xu, P. Mendes, U. Kummer, Bioinformatics 2006, 22, 3067-3074.

[3] T. Bäck, H.-P. Schwefel, Evolutionary Computation 1993, 1, 1-23.

[4] J. A. Nelder, R. Mead, The Computer Journal 1965, 7, 308-313.

[5] R. Seneviratne, S. Khan, E. Moscrop, M. Rappolt, S. P. Muench, J. C. Jeuken, P. A. Beales, Methods 2018, 147, 142-149.

[6] aW. F. Paxton, D. Price, N. J. Richardson, Soft Matter 2013, 9, 11295-11302; bS. Y. Yu, T. Azzam, I. Rouiller, A. Eisenberg, J. Am. Chem. Spc. 2009, 131, 10557-10566; cN. R. Clement, J. M. Gould, Biochem. 1981, 20, 1534-1538; dJ.-L. R. M. Seigneuret, Biochem. 1986, 25, 6723-6730; eC. L. Kuyper, J. S. Kuo, S. A. Mutch, D. T. Chiu, J. Am. Chem. Soc. 2006, 128, 3233-3240.

[7] D. W. Deamer, J. Bioenerg. Biomembr. 1987, 19, 457-479.
